# Anti-tumor memory CD4 and CD8 T-cells quantified by bulk T-cell receptor (TCR) clonal analysis

**DOI:** 10.3389/fimmu.2023.1137054

**Published:** 2023-03-23

**Authors:** Yanhua Gao, Ira Bergman

**Affiliations:** ^1^ Department of Pediatrics, Children's Hospital of Pittsburgh, University of Pittsburgh School of Medicine, Pittsburgh, PA, United States; ^2^ Department of Neurology, University of Pittsburgh School of Medicine, Pittsburgh, PA, United States; ^3^ Department of Immunology, University of Pittsburgh School of Medicine, Pittsburgh, PA, United States

**Keywords:** memory T cells, T-cell receptor clonotypes, immune repertoire analysis, tumor vaccination, clonotype analysis

## Abstract

Simple, reliable methods to detect anti-tumor memory T-cells are necessary to develop a clinical tumor vaccination program. A mouse model of curative viral onco-immunotherapy found that peritoneal tumor challenge following cure identified an oligoclonal anti-tumor memory CD4 and CD8 T-cell response. Clonotypes differed among the challenged animals but were congruent in blood, spleen and peritoneal cells (PC) of the same animal. Adoptive transfer demonstrated that the high-frequency responding T-cells were tumor specific. Tetramer analysis confirmed that clonotype frequency determined by T-cell receptor (TCR)- chain (TRB) analysis closely approximated cell clone frequency. The mean frequency of resting anti-tumor memory CD4 T-cells in unchallenged spleen was 0.028% and of memory CD8 T-cells was 0.11% which was not high enough to distinguish them from background. Stimulation produced a mean ~10-fold increase in splenic and 100-fold increase in peritoneal anti-tumor memory T-cell clonotypes. This methodology can be developed to use blood and tissue sampling to rapidly quantify the effectiveness of a tumor vaccine or any vaccine generating therapeutic T-cells.

## Introduction

Vaccination to generate antibody-mediated protection from infectious diseases has been a spectacularly successful, inexpensive, public health measure, rivaled in effectiveness only by cleanliness of water and society. T-cells, the alternative effector of the adaptive immune system, have not been harnessed to prevent human disease. The only possible exception may be BCG vaccination which, in some populations, has reduced the incidence of severe tuberculosis (1). One reason for this failure is the lack of a simple, reliable and valid measure of T-cell memory (2). EliSpot is widely used but long incubations with multiple cell types and biologic reagents make it a complex procedure that is difficult to standardize. Validation of high EliSpot response with clinical effectiveness has not been consistently shown. Tetramer analysis, once the tetramer is compounded, is simple and reliable but not available for almost any CD4 antigens and only a small number of CD8 antigens. The lack of a quantifiable memory T-cell response prevents incremental progress in vaccine development because the only outcome measure is clinical success or failure. The goal of this project was to use recent developments in next generation sequencing (NGS) and TCR clonal analysis to develop a simple, easily standardized measure of anti-tumor CD4 and CD8 memory T-cell response.

## Materials and methods

### Cells, antibodies, chemicals and animals

D2F2/E2 cells, a mouse mammary tumor line that has been stably transfected with a vector expressing the human HER2/neu gene and its parent cell line, D2F2 were a generous gift from Dr. Wei-Zen Wei, (Karmanos Cancer Institute, Wayne State University, Detroit, MI USA). Early passage cells were frozen and periodically thawed for experimental use or restocking. Mycoplasma testing was negative using the Impact III PCR profile from IDEXX (RADIL, Columbia, MO, USA). Anti-CTLA4 monoclonal antibody (mAb 9H10) was obtained commercially (BioXcell Fermentation/Purification Services #BE0131, West Lebanon, NH, USA) as was cyclophosphamide (Bristol-Myers Squibb Co., Princeton, NJ, USA). Mice were 8 to 20 weeks of age and weighed 20-25 g. Thy 1.2 BALB/c were obtained from Taconic (Hudson, NY). Animal studies were approved by the University of Pittsburgh institutional Animal Care and Use Committee (IACUC Protocol #: 21028761).

### Replicating recombinant vesicular stomatitis virus (rrVSV)

A replicating virus expressing the following properties was created from vector components as previously described (3): Preferential infection of cells expressing human HER2/neu, expression of mouse granulocyte-macrophage colony-stimulating factor, and expression of enhanced green fluorescent protein. Construction used vectors generously supplied by Dr. John K. Rose (Department of Pathology, Yale University, New Haven, CT, USA) and Genentech Inc., South San Francisco, CA, USA).

### Tumor implants and treatment

Female BALB/c mice weighing 20-25g and aged 8-20 weeks were implanted intraperitoneally (IP) with 2 x 10^6^ D2F2/E2 cells in 300 µl PBS. Viral immune-oncotherapy consisted of treatment on day 3 after peritoneal implant with IP rrVSV, 1 x 10^8^ ID, on day 4 with 200 µg anti-CTLA4 mAb IP and on day 5 with cyclophosphamide (CTX), ~100 mg/kg IP. Cure was defined by survival without any symptoms of disease for >100 days after tumor cell implantation. Control mice received the same viral immune-oncotherapy but were never implanted with tumor.

### Memory T-cells

Memory T-cells were obtained from spleens, peritoneal cells or blood of cured animals and controls. Animals were sacrificed prior to spleen cell harvesting. Spleens were harvested, minced and ground through a 70 µM nylon cell strainer (#352350, BD Falcon, Franklin Lakes, NJ). Red blood cells were removed using RBC lysis buffer (Alfa Aesar, J62150AP). Peritoneal washings were performed by injecting 10 ml of sterile PBS into the peritoneum through a 16-gauge needle which was left in place. Two minutes later all the fluid that could be aspirated easily into the syringe was collected, ~ 9 ml. All cells were washed twice with PBS and re-suspended in PBS. Peripheral blood was collected by submandibular punch and dripping the blood into a heparinized tube. Mononuclear cells were separated using Lymphocytes Separation Medium. CD4 and CD8 T-cells were isolated by flow cytometry (see below).

### Adoptive therapy and tumor challenge

Peritoneal tumors were established in host animals and treated 3 days later by adoptive transfer of splenocytes from cured animals. As previously described, host animals were pre-treated with cyclophosphamide one day before transfer of memory cells (4). Tumor challenge to cured or control mice was achieved by injecting 2 x 10^6^ D2F2/E2 cells IP.

### Flow cytometry

Peritoneal cells were suspended in ice-cold PBS/0.1% BSA/0.2% Azide and stained with combinations of the following antibodies: CD4- APC-eFluor 780 (eBioscience 47-0041-82), CD8a- PE-Cyanine7 (eBioscience 25-0081-82), CD90.2-PE (BD 553006), Live-Dead fixable red Kit (Life Science, NC0836452) and H-2K(d)/TYLPTNASL, human HER2 p63 tetramer conjugated with APC (NIH Tetramer Core Facility at Emory University). Immunofluorescence was quantified using a LSR Fortesa (Becton Dickinson, Mountainview, CA, USA) and cell sorting was performed using a FACSAria II machine (Becton Dickinson) (5).

### RNA extraction

RNA extraction was completed using Qiagen’s RNeasy Plus Micro kit (Qiagen:74034), according to the manufacturer’s instructions with DNA elimination columns. Briefly, cells were pelleted in a centrifuge at 4°C for 8 minutes at 800xg, then lysed in RLT buffer with beta- mercaptoethanol. RNA was eluted with 22 µl of RNase/DNase free H_2_0. RNA quality was assessed using an Agilent HS RNA ScreenTape (Agilent: 5067-5579) on an Agilent 2200 TapeStation. RNA concentration was quantified with a Qubit HS RNA assay kit (Invitrogen: Q32855) on a Qubit 4 (Invitrogen: Q33238).

### TCR-seq library generation

Libraries were generated with the Takara SMARTer Mouse TCR a/b profiling kit (Takara: 634403) according to the manufacturer’s instructions. Twenty-one cycles were used for PCR1, after which 1 μl of product was used for indexing PCR2 with 19 cycles. Samples with RNA concentrations ≤ 1.0 ng/µl RNA used 22 cycles for PCR1, after which 2 μl of product was used for indexing PCR2 with 19 cycles. Library assessment and quantification was done using Qubit 1x HS DNA assay kit (Invitrogen: Q33231) on a Qubit 4 fluorometer, and a HS NGS Fragment kit (Agilent: DNF-474-1000) on an Agilent 5300 Fragment Analyzer. Libraries were normalized and pooled by calculating the nM concentration based off the fragment size (base pairs) and the concentration (ng/μl) of the libraries.

### TCR-seq library sequencing

Sequencing was performed on an Illumina MiSeq using a MiSeq 600 v3 flow cell (Illumina: MS-102-3003). The pooled library was loaded at 13.5nM with 10% PhiX, generating 2x300 bp paired-end reads. Library generation and sequencing was performed by the University of Pittsburgh Health Sciences Sequencing Core (HSSC), Rangos Research Center, UPMC Children’s Hospital of Pittsburgh, Pittsburgh, Pennsylvania, United States of America.

### TCR repertoire analysis

MiXCR (platform 3.0.13; MiLaboratories Inc, Sunnyvale, CA, USA) was used to align and assemble the raw paired-end Fastq files sequencing reads and assemble identical and homologous reads into clonotypes, correcting for PCR and sequencing errors. The program provided detailed information for each clone, including fractions, counts, nucleotide sequence, and amino acid sequence, as well as alignments for multiple samples, repertoire overlap analysis, diversity estimation and segment usage (6). Mouse NKT TRA clonotypes, identified by the canonical CDR3 sequence, CVVGDRGSALGRLHF, were excluded from analysis (7, 8) as were mouse MAIT TRA clonotypes, identified by the canonical CDR3 sequence, CAVRDSNYQLIW (9). This methodology does not exclude TRB clonotypes associate with NKT or MAIT cells but the NKT TRA clonotype was never found at high frequency in peritoneal cells following tumor stimulation indicating that the high frequency response did not contain any NKT cells. Mouse MAIT TRA clonotypes were never found at high frequency in any sample suggesting that these cells were not a confounder in this work. Finally, results for TRA clonotypes, which have definitely excluded NKT and MAIT cells are provided for all analysis and do not change any of the conclusions.

### Statistics

GraphPad Prism version 7.0 (GraphPad Software, Inc., San Diego, CA, USA) was used for presentation and statistical analysis of the data.

## Results

### Tumor challenge in cured mice stimulates a large oligoclonal CD4 and CD8 memory T-cell response in spleen cells

**Figure 1:**
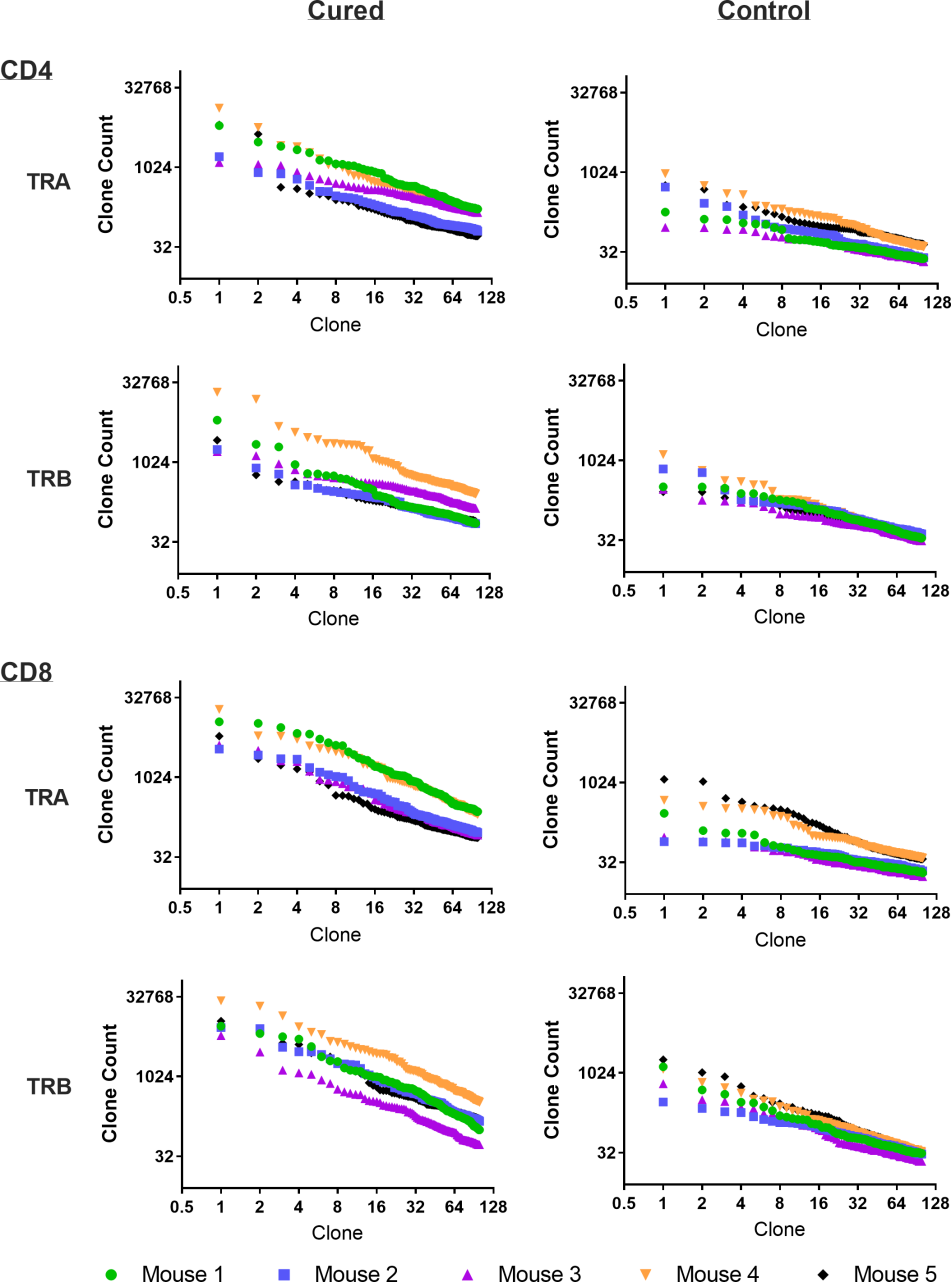
Frequency analysis of tumor-challenged spleen T-cells. Intraperitoneal tumor challenge in mice who had received rrVSV oncolytic immunotherapy to cure implanted tumor (tumor-cured mice) compared to mice with full viral therapy but no tumor implant (virus control mice). Splenic CD4 and CD8 T-cells were harvested 5 days after challenge and TCR clones quantified. The top 100 clones are plotted and results shown separately for CD4 and CD8 T-cells and TCR-α (TRA) and TCR-β (TRB) receptors (Log2 scale for X and Y axes).

**Figure 2:**
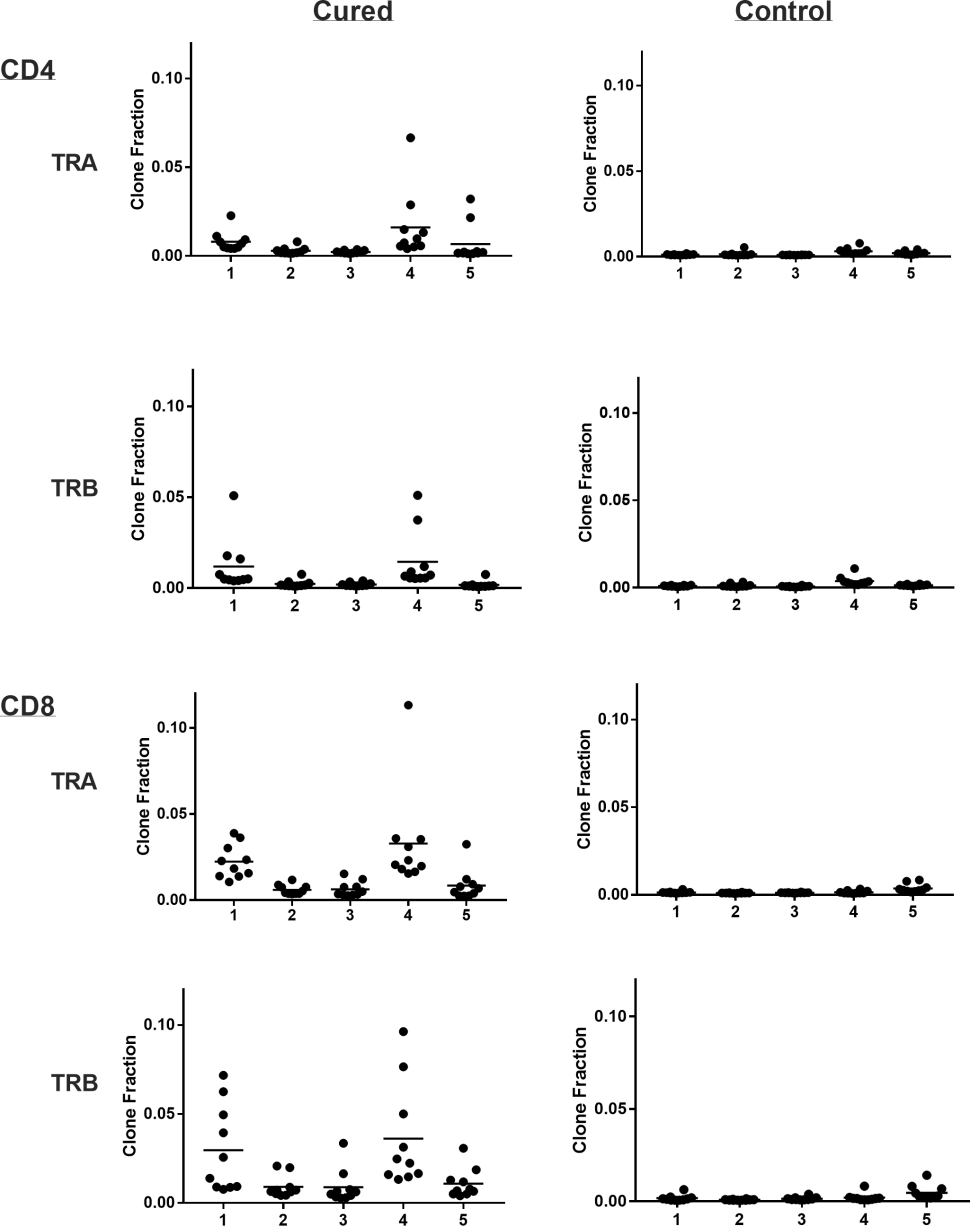
Clone frequency following challenge in spleens of tumor-cured mice compared with virus control mice. Scatter plot of the top 10 clones. Results are shown separately for CD4 and CD8 T-cells; TRA and TRB. Each number represents a single cured or control mouse.

**Figure 3:**
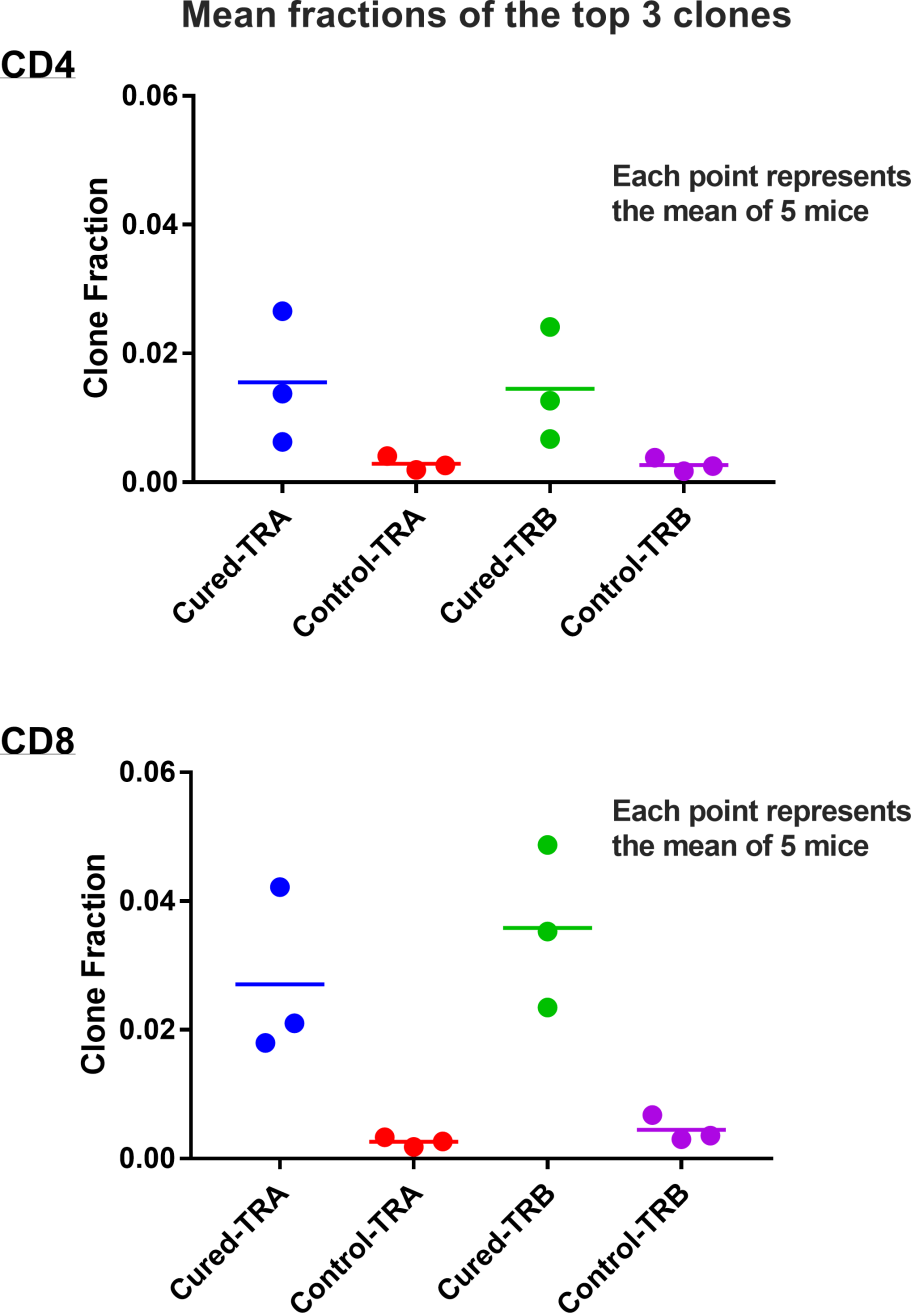
Mean fractions of the responding clones in spleens of tumor-cured mice compared with virus control mice. Scatter plot of the top 3 clones. Results are shown separately for CD4 and CD8 T-cells and TRA and TRB receptors. The mean fractions of the top 3 clones were 5.4-fold higher for CD4 T-cells and 8-10 fold higher in CD8 T-cells in tumor-cured mice compared with virus control mice (all difference were significantly different; p=0.05 and 0.04 for TRA and TRB CD4 T-cells, and p= 0.0164 and 0.0066 for TRA and TRB CD8 T-cells; unpaired, one-tailed t test; n=5 for each group).

Viral immuno-oncotherapy consisting of a replicating recombinant VSV (rrVSV) targeted to Her2, and single doses of anti-CTLA4 Mab and cyclophosphamide cures 3 day implanted Her2+ peritoneal tumors in Balb/c mice. Cure is immunologically dependent, requires both CD4 and CD8 T-cells and generates memory T-cells by 90 days after treatment (5, 10, 11). The anti-tumor memory response was assessed by re-challenging tumor-cured mice with IP administration of tumor cells. TCR clonal analysis, TCRα (TRA) and TCRβ (TRB), was performed on spleen cells harvested 5 days after challenge. A control group consisted of animals who received targeted rrVSV oncolytic immunotherapy, at least 100 days prior to challenge but were never implanted with tumor (virus control mice). All animals in the tumor- cured group showed an oligoclonal set of high-responding splenic TCR clones for CD4 and CD8 T-cells that was not observed in the control group (**Figures 1**–**3**). The mean clone fractions of the top 3 clones was 5-fold higher for CD4 T-cells and 8-10 fold higher in CD8 T-cells in tumor-cured mice compared with virus control mice (**Figure 3**). The high responder response to challenge varied among the cured animals but was uniformly low among the control animals (**Figure 2**). At least one high frequency clone was higher than the highest control value in 5/5 mice (100%) for CD8 T-cells and 4/5 mice (80%) for CD4 T-cells (**Figure 2**). This highest frequency oligoclonal response in spleen varied from 2-18 CD8 and 0-5 CD4 T-cell anti-tumor TRA or TRB clonotypes per animal (median CD8 = 2.5 and CD4 = 1.5).

### The oligoclonal high-responder spleen response to tumor challenge reflects the peritoneal anti-tumor response and is distinct for each animal

Our previous work in this model system has shown that anti-tumor memory CD4 and CD8 T-cells accumulate in the peritoneum following IP tumor challenge in tumor-cured mice (5, 11). In this study, the peritoneal cell response to tumor challenge in experimental and control groups is displayed in **Supplemental Figure S1**. The specific migration of anti-tumor memory T-cells to the peritoneum following stimulation was supported by the absence of high frequency peritoneal NKT cells, which could easily be identified by an invariant clonotype and were abundant in the spleens of experimental and control animals (12). Clonal overlap was determined between the spleen and peritoneal response in the same animals to show that the anti-tumor response seen in the peritoneum was reflected in the spleen, providing evidence that the spleen response was an anti-tumor response (**Table 1**). In total, 70% of high-responding T-cells in the peritoneum were also high-responders in the spleen (62.5% of the CD4 and 82% of the CD8 T-cells). Each animal with a high-responding clonotype in the peritoneum had at least one identical high-responding clonotype in the spleen. The spleen response was therefore an excellent surrogate for the peritoneal response.

**Table 1:** Comparison of high-responding clones in the spleen and peritoneum of the same animal.

Number of high responding peritoneal clones and number of identical clones in spleen*		
	PC	Spleen
CD4	16	11
CD8	11	9

*n=5 animals except for peritoneal CD8 T-cells which were inadequate for testing on one animal. Clonotypes were considered high-responders when their frequency was greater than the mean of the top 2 clones, of the same T-cell category, in the virus-control group.

**Figure 4:**
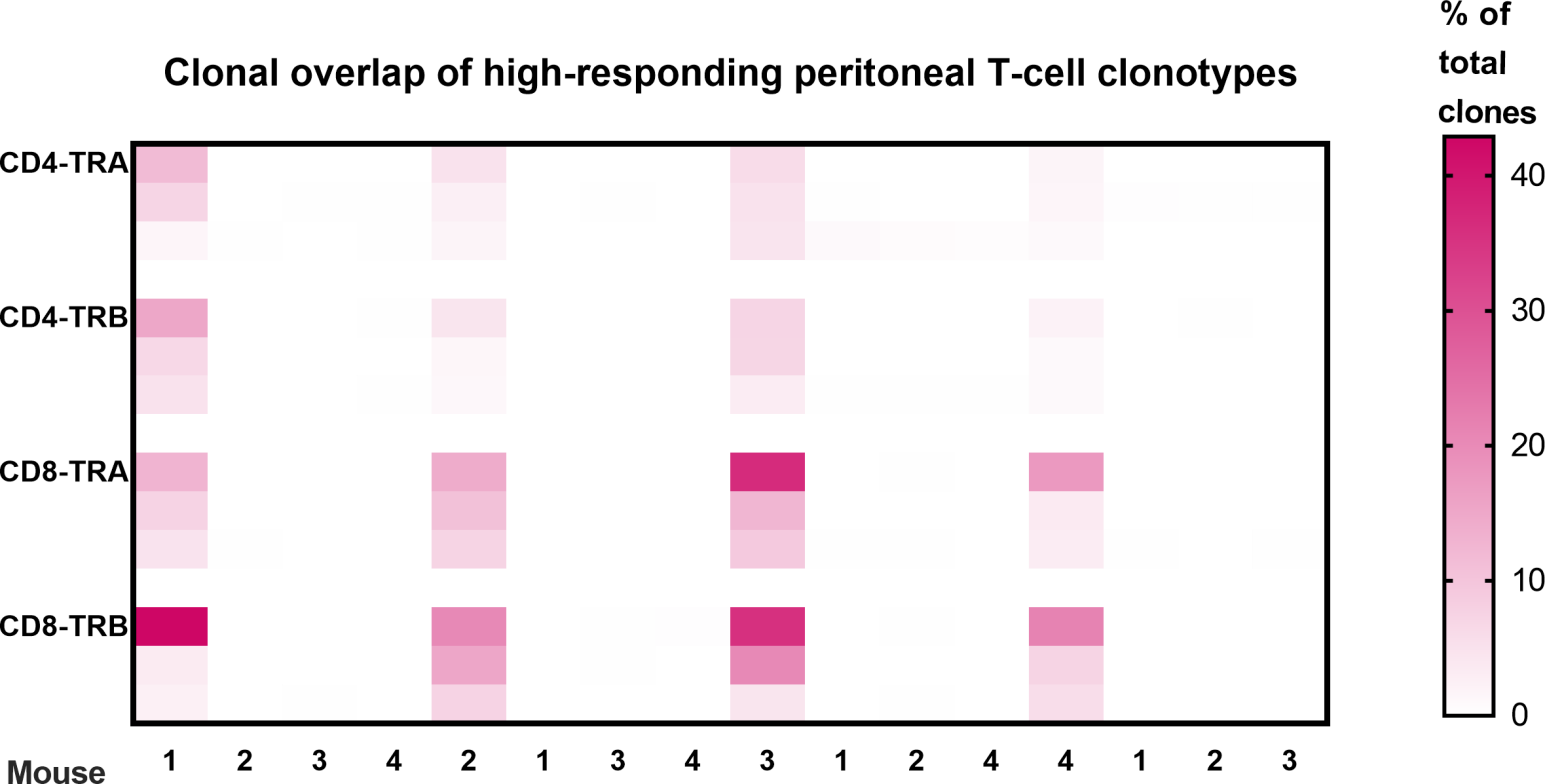
Clonal overlap of high-responding peritoneal T-cell clonotypes. The top 3 responding clonotypes in each animal were the index clones for heatmap comparison with each of the other animals. There is no overlap of high-frequency T-cell clonotypes in peritoneal cells from different animals. Clonotypes were considered peritoneal high-responders when their frequency was greater than the mean of the top 2 clones in the virus-control group. (Scale is % of total clones; n=4; a 5^th^ animal was excluded because collection of peritoneal CD8 T-cells was inadequate for analysis.).

Clonal overlap was also determined between the peritoneal cell response among the different animals (**Figure 4**). These results show that none of the highest-responder clonotypes in one animal were also high-responders in a different animal. As illustrated in **Figure 4**, some of the high-responder clonotypes were found in other animals but only at low frequency. Basically, each animal had a private set of high-frequency tumor-responding CD4 and CD8 TCR clonotypes.

### Tetramer analysis demonstrates that high responder peritoneal CD8 T-cells were anti-tumor memory T-cells and had distinct clonotypes for each animal

**Figure 5:**
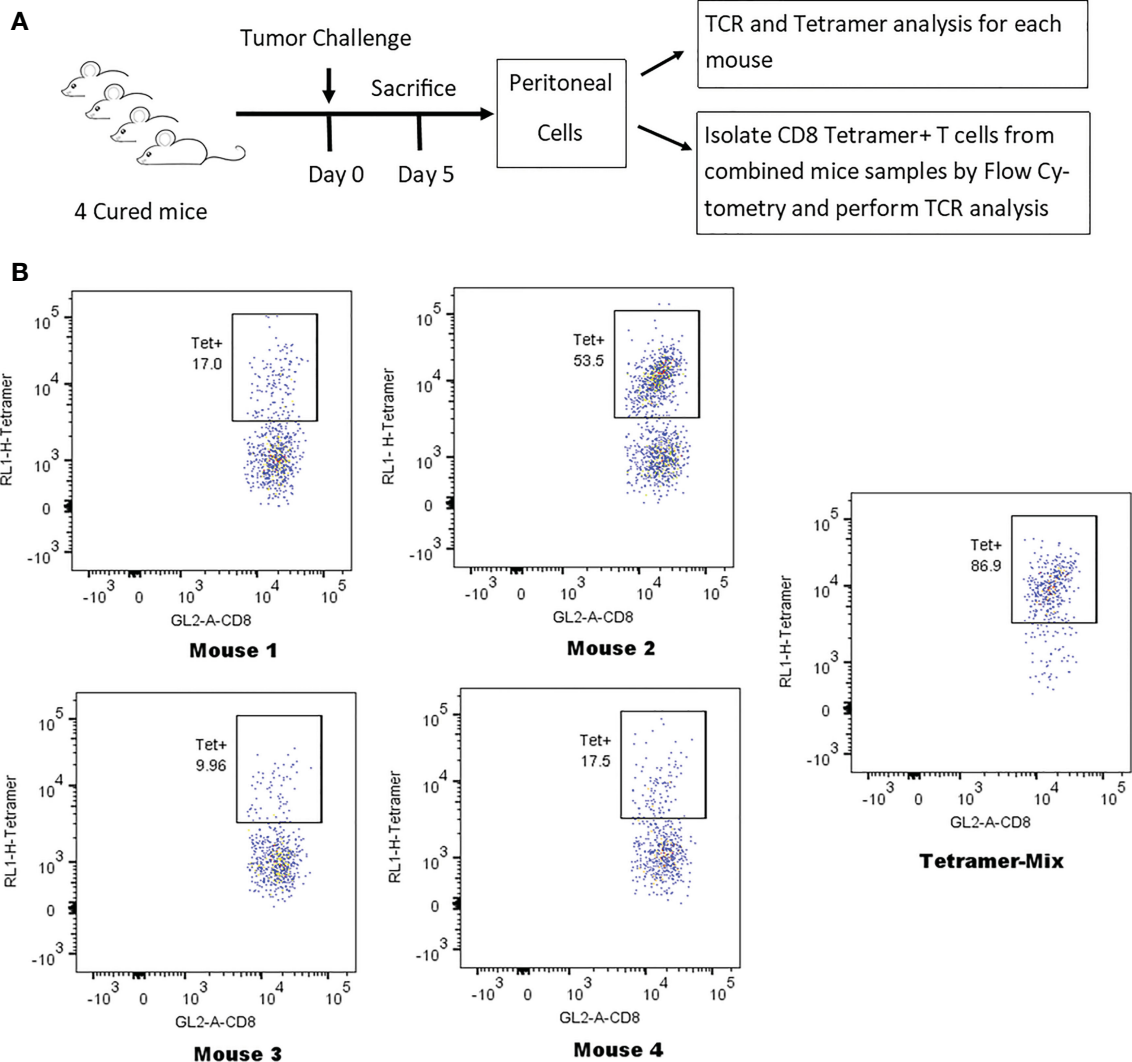
Identification of tetramer+ clonotypes within the total CD8 T-cell population. **(A)** Illustration of experiment identifying TCR clones of tetramer+ CD8 T-cells. **(B)** Anti-tumor memory CD8 T-cells identified by flow cytometry following staining with the human HER2 p63 tetramer conjugated with APC. The percent tetramer-positive cells is noted for the peritoneal cells of each individual mouse and for the tetramer-positive mix from all 4 mice.

Tumor-cured mice (n=4) received peritoneal tumor challenge followed 5 days later by isolation of CD8 T-cells from peritoneal lavage. Each sample was divided in half. One half from each animal had flow cytometry tetramer analysis and TCR clonotyping performed individually for each animal. The other individual samples were combined and tetramer+ CD8 T-cells were identified and separated by flow cytometry. Flow cytometry tetramer analysis and TCR clonotyping was then performed on this mixed sample from 4 mice (**Figure 5**). A combined sample of tetramer+ CD8 T-cells was required to assure an adequate concentration of mRNA for TCR clonotyping analysis. Purity of the tetramer+ mixture was 86.9% and the delineation between tetramer-positive and tetramer-negative cells with flow cytometry was not absolute (**Figure 5B**), leading to false positive clonotypes in the tetramer+ mixture. True positives were determined by using all high-frequency clonotypes (>1%) in the individual animals as index cases, determining the frequencies of the identical clonotypes in the tetramer+ mix and plotting the inverse ratio (**Supplemental Figure S2**). High ratios were easily separated from low ratios and had similar patterns in TRA and TRB for each animal. Clonotypes with ratios 0.18 were considered true positives.

**Figure 6:**
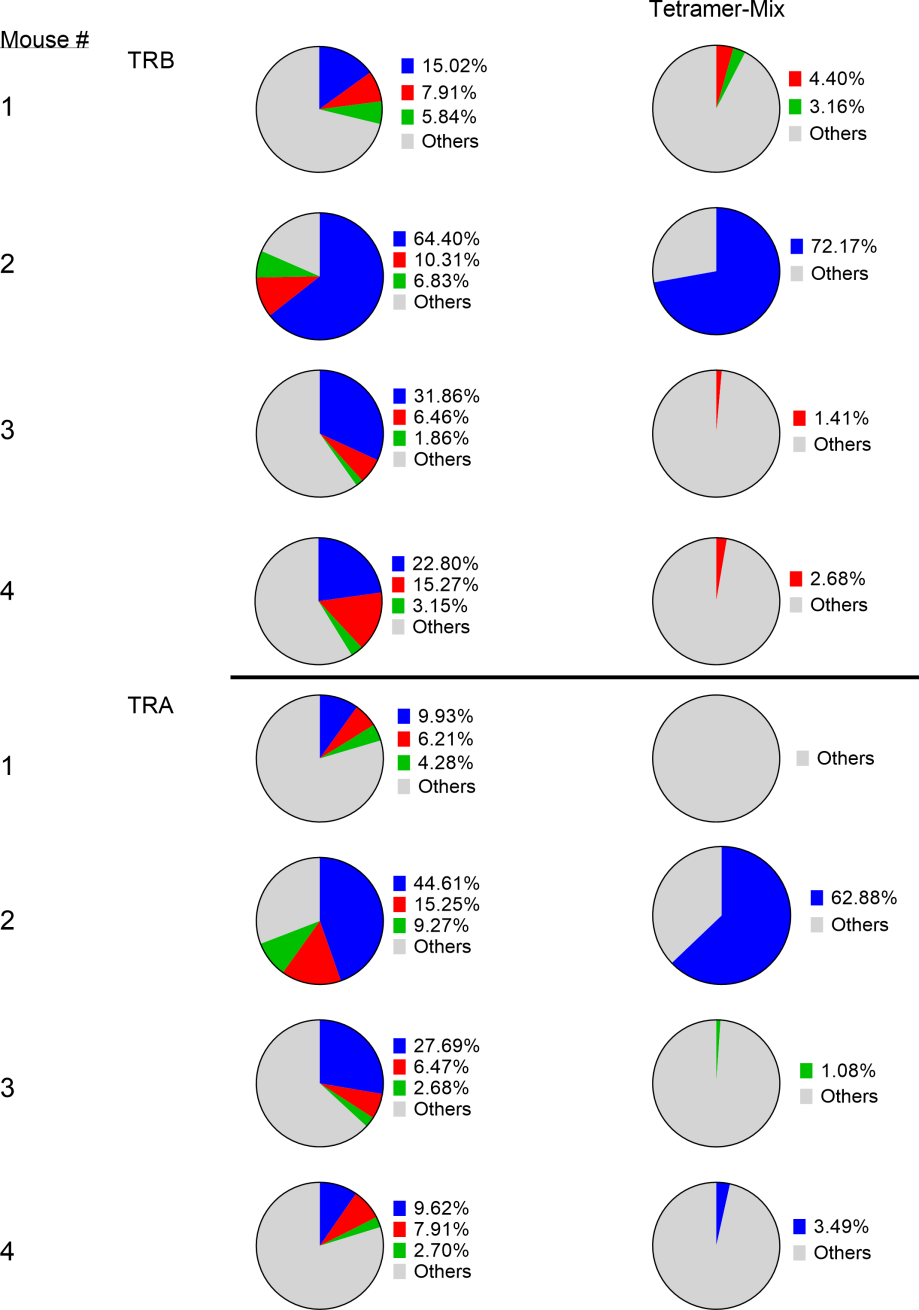
Identification of tetramer^+^ clonotypes within the total CD8 T-cell population. The left column displays the top 3 CD8 T-cell clonotypes in peritoneum in 4 cured animals challenged with IP tumor cells. The right column displays matching TCR clonotypes in the tetramer+ mix from the 4 animals. The blue, red and green colors represent the top 3 clones in each animal and are unique high-frequency clonotypes in each animal.

**Figure 7:**
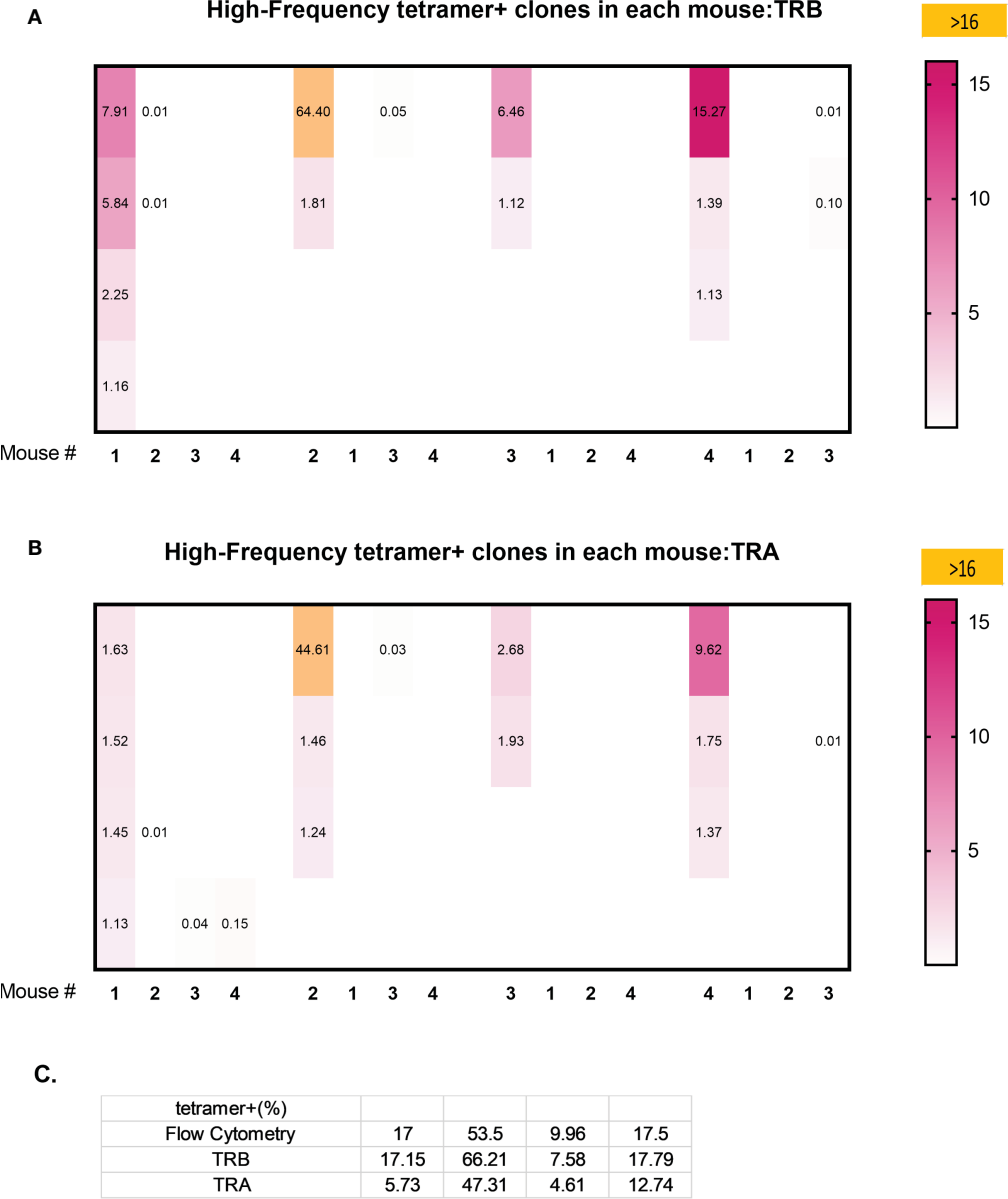
Clonal overlap of high frequency tetramer+ CD8 T-cells clonotypes among 4 animals. Tetramer+ CD8 T-cells from each mouse (the first column in each set) was heatmap compared with the top 20 responding clonotypes in each of the other mice. **(A)** TRB clonotypes. **(B)** TRA clonotypes. **(C)** A comparison of the percentage of high frequency tetramer+ CD8 T-cells identified by fluorescence activated cell sorting (FACS) and by TRB or TRA clonotype analyis.

Analysis of the 3 highest clonotypes in each animal showed that 5/12 TRB clonotypes and 3/12 TRA clonotypes were tetramer+ (**Figure 6**). None of these 8 highest frequency responding tetramer+ clonotypes had the same TCR peptide sequence and, in fact each of the 24 highest frequency responding clonotypes had a unique sequence (13). An analysis of all tetramer+ clonotypes found 11 TRB clonotypes and 12 TRA clonotypes with frequency > 1% (**Figure 7**). The number of these tetramer+ clonotypes per mouse varied from 2 to 4 (median = 3) and each clonotype was unique (**Figure 7**). The same clonotype was sometimes found in other animals (5 TRA and 5 TRB) but never at frequency >0.15% and usually much lower (**Figure 7**). In summary, each animal had its own private high-frequency tetramer+ sequence and some had more than one. An unexpected finding was that tetramer+ frequency based on TRB clonotype frequency closely matched clone frequency results based on flow cytometry (**Figure 7**). TRA frequency was not as closely matched probably because ~30% of T-cells express 2 functionally rearranged TRA mRNAs (14, 15).

### Transfer experiments demonstrate that high responder splenic CD4 and CD8 T-cells were anti-tumor memory T-cells

**Figure 8:**
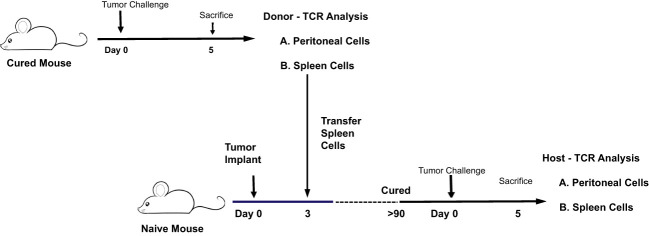
Illustration of experiment comparing the CD4 and CD8 TCR clonotype response to IP tumor challenge in donor animals cured of implanted tumor by rrVSV oncolytic immunotherapy and host animals cured by T-cell transfer from the donor animals.

**Figure 9:**
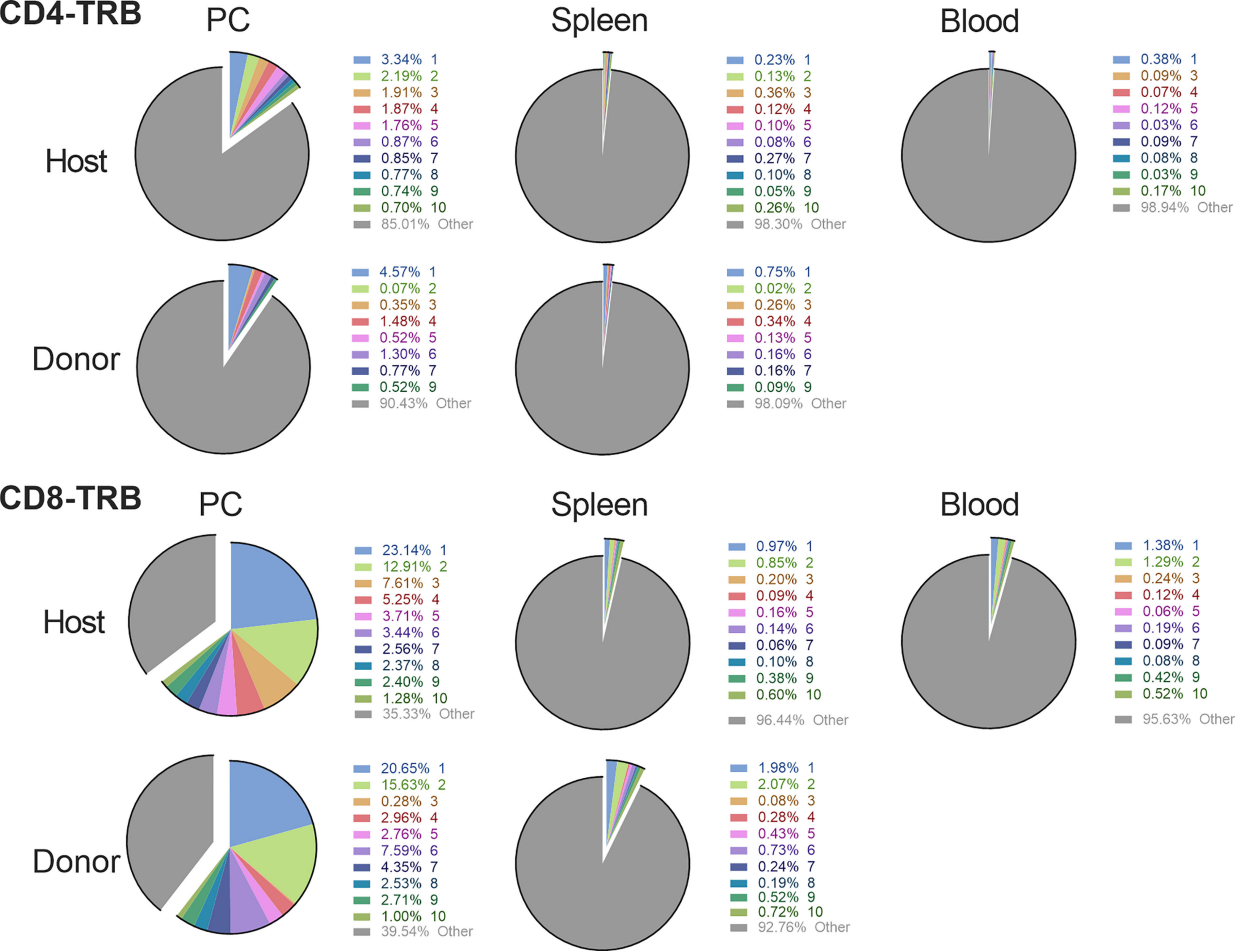
Comparison of the high frequency clonotypes following challenge in donor animals cured of implanted tumor by rrVSV oncolytic immunotherapy and host animals cured by T-cell transfer from the donor animals. Graphs show CD4 and CD8 T-cell TRB data from one representative pair of animals of three total pairs. The top 10 most frequent clones in the host peritoneal cells are the index clones and are compared with the donor peritoneal and spleen cells and the host spleen and blood cells. Identical numbers and colors within the CD4 and CD8 sets refer to identical clonotypes.

The CD4 and CD8 TCR clonotype response in spleen and peritoneum to IP tumor challenge was compared in donor animals cured of implanted tumor by rrVSV oncolytic immunotherapy and host animals cured by T-cell transfer from the donor animals (**Figure 8**). The critical finding was that the same high-responding TCR clonotypes were found in all 4 samples (**Figure 9** for TRB and **Supplemental Figure S3** for TRA), strongly suggesting that they represented anti-tumor memory clones. The high responder peritoneal CD4 and CD8 T-cells in the host animals were derived from the donor mice because they had matching clonotypes. Independent clones arising from the hosts would have had their own private sequences as shown in the previous sections. These were the therapeutic T-cells because cure was obtained solely by transfer of donor spleen cells. These cells were first recognized by IP challenge in the donor mice demonstrating that a high clonotype response to challenge is a reliable method to identify anti-tumor CD4 and CD8 T-cell clones. Peritoneal clonotypes were classified as definite, active anti-tumor memory if they appeared in both donor and host peritoneal cells at a frequency greater than the mean of the top 10 clonotypes from all control animals (**Figure 9**). The number of unique high-frequency anti-tumor memory TRB clonotypes in the peritoneum varied from 1 to 8 in CD4 and 4-8 in CD8 T-cells with a median of 4 CD4 T-cells and 6 CD8 T-cells. Donor spleen and host peritoneal clonotypes were also classified as definite, active anti-tumor memory if they appeared in both host PC and donor spleen at a frequency greater than the mean of the top 10 clonotypes from all control animals (**Figure 9**). The number of high-frequency unique anti-tumor memory TRB clonotypes in the donor spleen varied from 1 to 8 for CD4 and 5-7 for CD8 T-cells with a median of 3 CD4 T-cells and 6 CD8 T-cells. The frequency of anti-tumor CD4 TRB clonotypes was 12.7-fold higher in host peritoneum than host spleen and 7.9-fold higher for CD8 TRB clonotypes, confirming our previous work, that anti-tumor memory CD4 and CD8 T-cells migrate to and expand at the site of challenge in tumor-cured mice (5, 11). TRB clonotypes were used in these analyses because their frequency had the best correspondence with clone frequency, as determined above. Importantly, the anti-tumor memory clonotypes were also found in blood, at similar frequencies to spleen, indicating that these analyses may be practical clinically (**Figure 9** and **Supplemental Figure S3**).

### The mean resting frequency of anti-tumor memory T-cells in spleen is 0.028% for CD4 T-cells and 0.11% for CD8 T-cells

**Figure 10:**
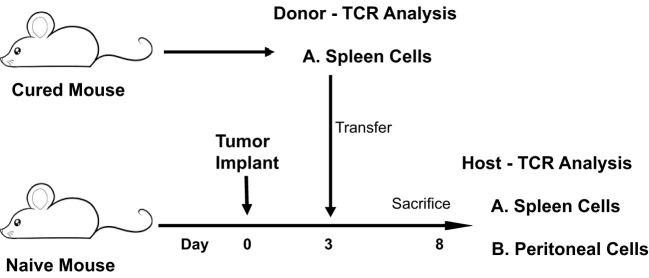
Identifying and determining the frequency of resting and challenged anti-tumor memory T-cells. Illustration of experiment comparing a TCR frequency analysis of memory anti-tumor T-cells in resting spleens of donor mice with challenged spleen and peritoneal T-cells of host mice.

**Figure 11:**
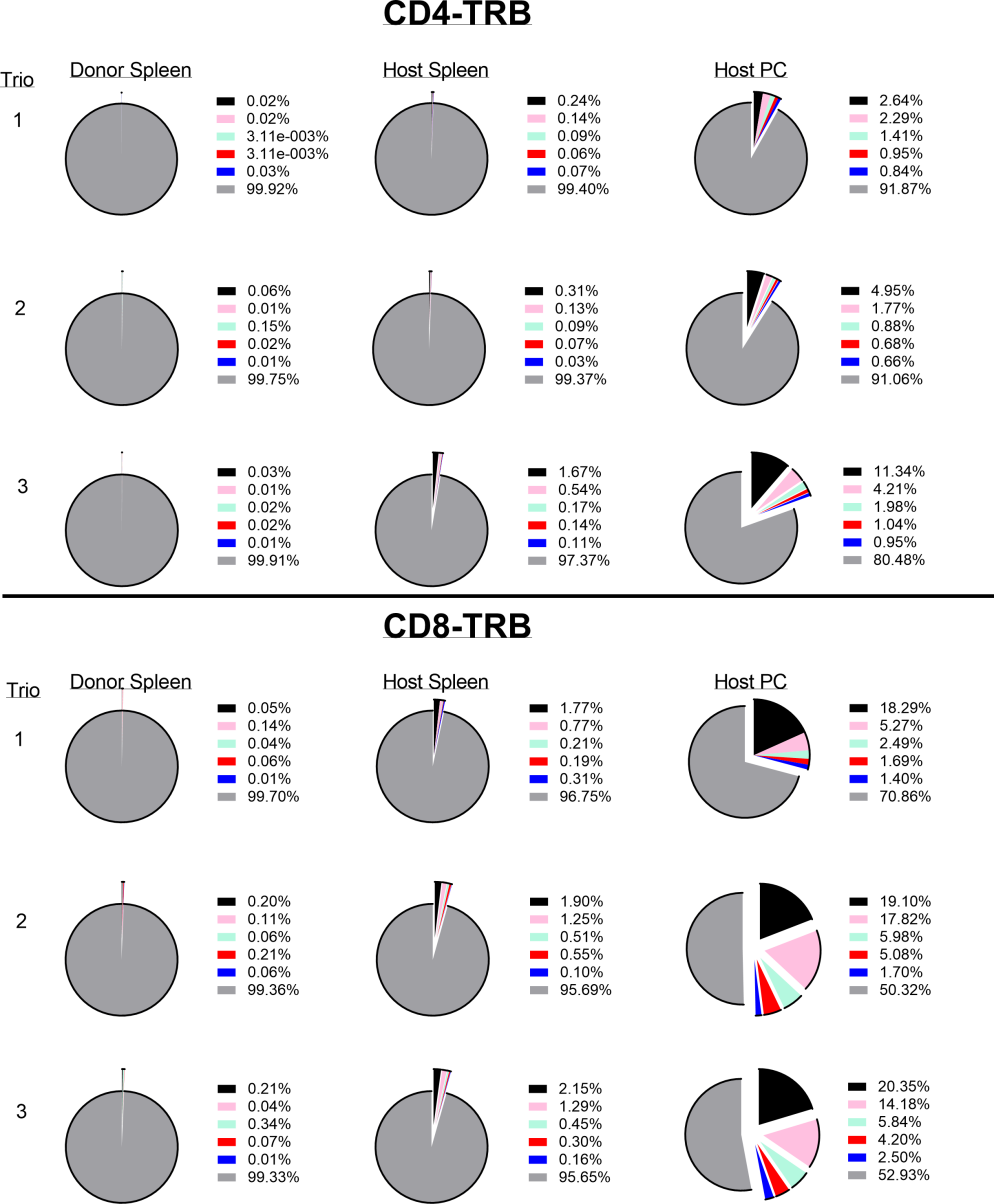
Identifying and determining the frequency of resting and challenged anti-tumor memory T-cells. The top 5 most frequent clones in the host challenged peritoneal T-cells are the index clones and are compared with the frequency of the same clones in the spleens of challenged host and unchallenged donor mice. Three trios of animals are shown in separate rows with the CD4 T-cells in the top half and the CD8 T-cells in the bottom half of the figure (TRB only). Identical numbers and colors within the CD4 and CD8 pairs refer to identical clonotypes.

**Figure 12:**
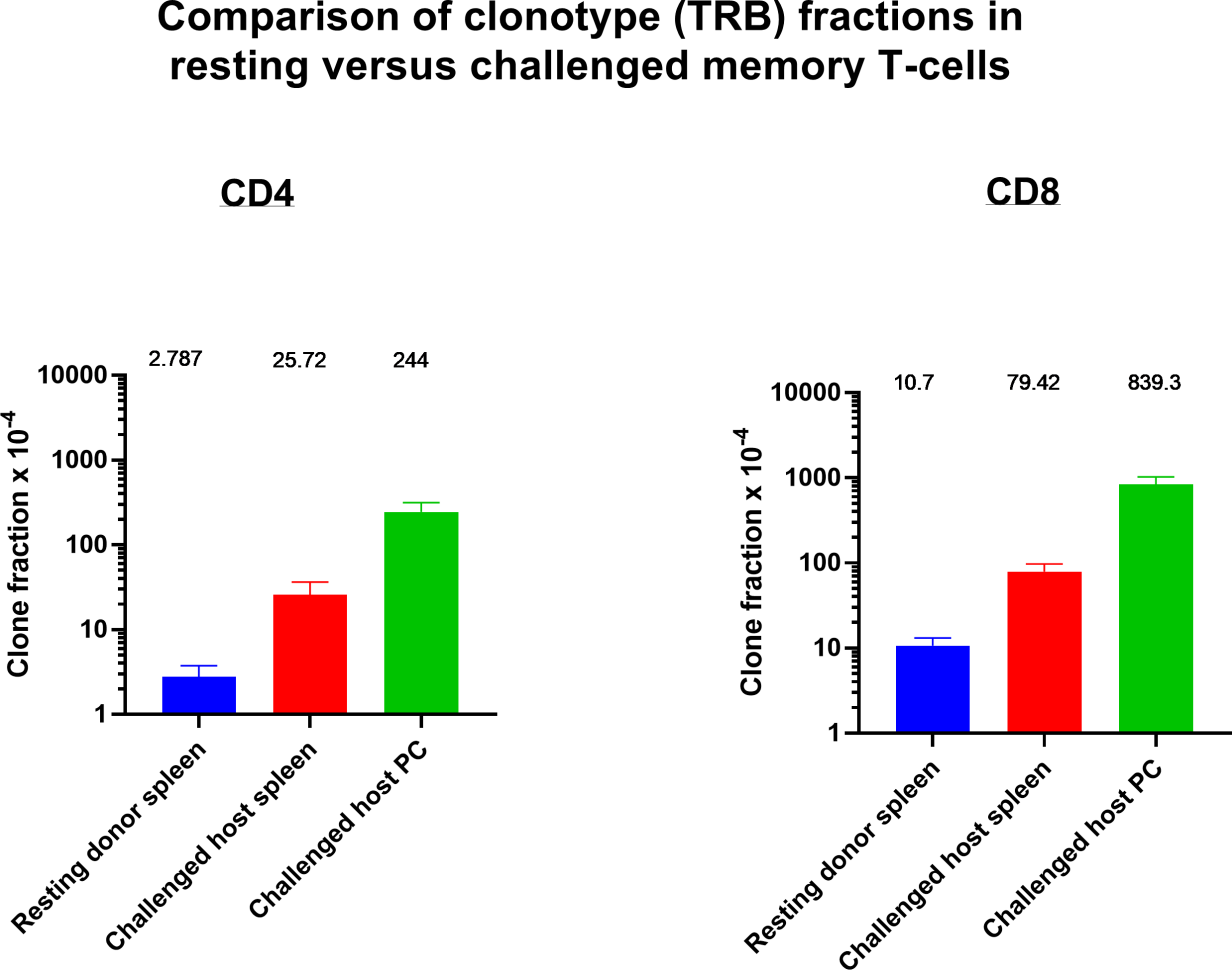
Identifying and determining the frequency of resting and challenged anti-tumor memory T-cells. The frequency of the top 5 anti-tumor memory T-cells in the peritoneum of host challenged mice are compared with the frequency of the same clones in the spleen of host challenged mice and the spleen of resting donor mice. (n=3 in each group, CD4 and CD8, TRB only); mean values and SEM bars above the column for each group).

The resting frequency of anti-tumor memory T-cells was determined by harvesting spleen cells from cured, unchallenged, resting, donor mice and assaying TCR clonotypes from one aliquot of 1 x 10^7^ cells. A second aliquot of 5 x 10^7^ spleen cells were challenged by transfer to a host animal with a 3 day implanted peritoneal tumor. Host spleen and peritoneal TCR clonotypes were assayed 5 days later (**Figure 10**). Clonotypes in the resting donor cured tumor mice that were identical to high frequency clonotypes in the peritoneum of challenged mice were anti-tumor memory clonotypes. Independent clones arising from the hosts would have had their own private sequences as shown in the previous sections. In addition, only memory cells from the donors and not naïve cells from the hosts, could be present at sufficient concentration and multiply rapidly enough in 5 days to produce the high frequency clones found in the peritoneum. Challenge of naïve animals with peritoneal tumor cells produced only low clonotype frequencies at 5 days (data not shown). Finally, our current and previous work has shown that spleens from donor mice cured of tumor by rrVSV oncolytic immunotherapy contain potent therapeutic anti-tumor memory T-cells (4, 5, 10, 11, 16, 17). Using the top 5 peritoneal clonotypes as index clonotypess, all CD4 and CD8 T-cell TRB clonotypes had identical clones in the donor unchallenged mice, although 2 CD4 clonotypes from one animal had notably lower resting frequencies than all the others (**Figure 11** for TRB and **Supplemental Figure S4** for TRA). The mean frequency of these anti-tumor memory T-cells in resting T-cells averaged 0.028% for CD4 T-cells and 0.11% for CD8 T-cells (**Figure 12**). The highest frequency clone for CD4 T-cells was 0.15% and for CD8 T-cells was 0.34%. These findings match well with tetramer-based studies of anti-viral human memory CD4 and CD8 T-cells and mouse CD4 T-cells but are 30-fold lower than several previous reports of mouse anti-viral memory CD8 T-cells. All studies agree that resting memory CD8 T-cells form a higher fraction of total CD8 T-cells than resting memory CD4 T-cells form of total CD4 T-cells (13, 18–24). TRA data was similar (**Supplemental Figure S4**) but we focused on TRB clonotypes as the best surrogate for TCR clones for several reasons: T-cells almost invariable express a single TRB mRNA but ~1/3 express 2 TRA mRNA (14, 15); the main contribution to TCR-peptide binding comes from the TRB CD3 sequence (25); and data presented above showed a close correspondence between TRB clonotype frequency and cell clonal frequency.

**Figure 13:**
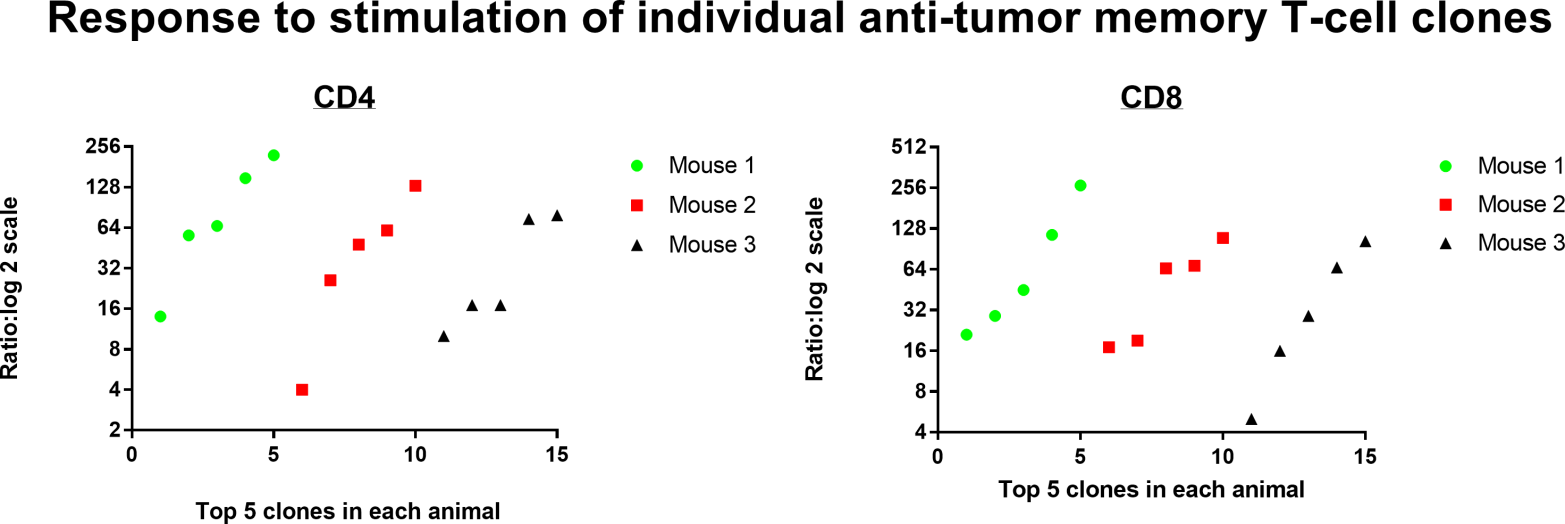
Response to stimulation of individual anti-tumor memory T-cell clones. The total number of memory T-cells in transferred donor spleen cells were compared with total number of stimulated host memory T-cells harvested from spleen and peritoneum. The top 5 most frequent CD4 and CD8 memory T-cell clones for each of 3 animals are plotted separately (TRB clonotypes). The mean increase in clone count was 65-fold for both CD4 and CD8 T-cells.

Resting donor spleen TRB clonotypes had ~ 1/10 the frequency of challenged memory clonotypes in spleen and ~1/100 the frequency of challenged memory clonotypes in the peritoneum (**Figure 12** for TRB and **Supplemental Figure S5** for TRA). CD4 T-cells increased from 0.028% to 0.26% to 2.44% and CD8 T-cells increased from 0.11% to 0.79% to 8.39%. The mean increase in TRB clonotype count from transferred donor T-cells to combined harvested spleen and peritoneal T-cells was 65-fold for both CD4 and CD8 memory T-cells (**Figure 13** for TRB and **Supplemental Figure S6** for TRA). This indicated a minimum mean number of 6 doublings in 5 days because potential anti-tumor T-cells in other tissues such as lymph nodes, bone marrow and blood were not harvested. Doublings in individual clones varied from 2 to 8 (**Figure 13**).

**Figure 14:**
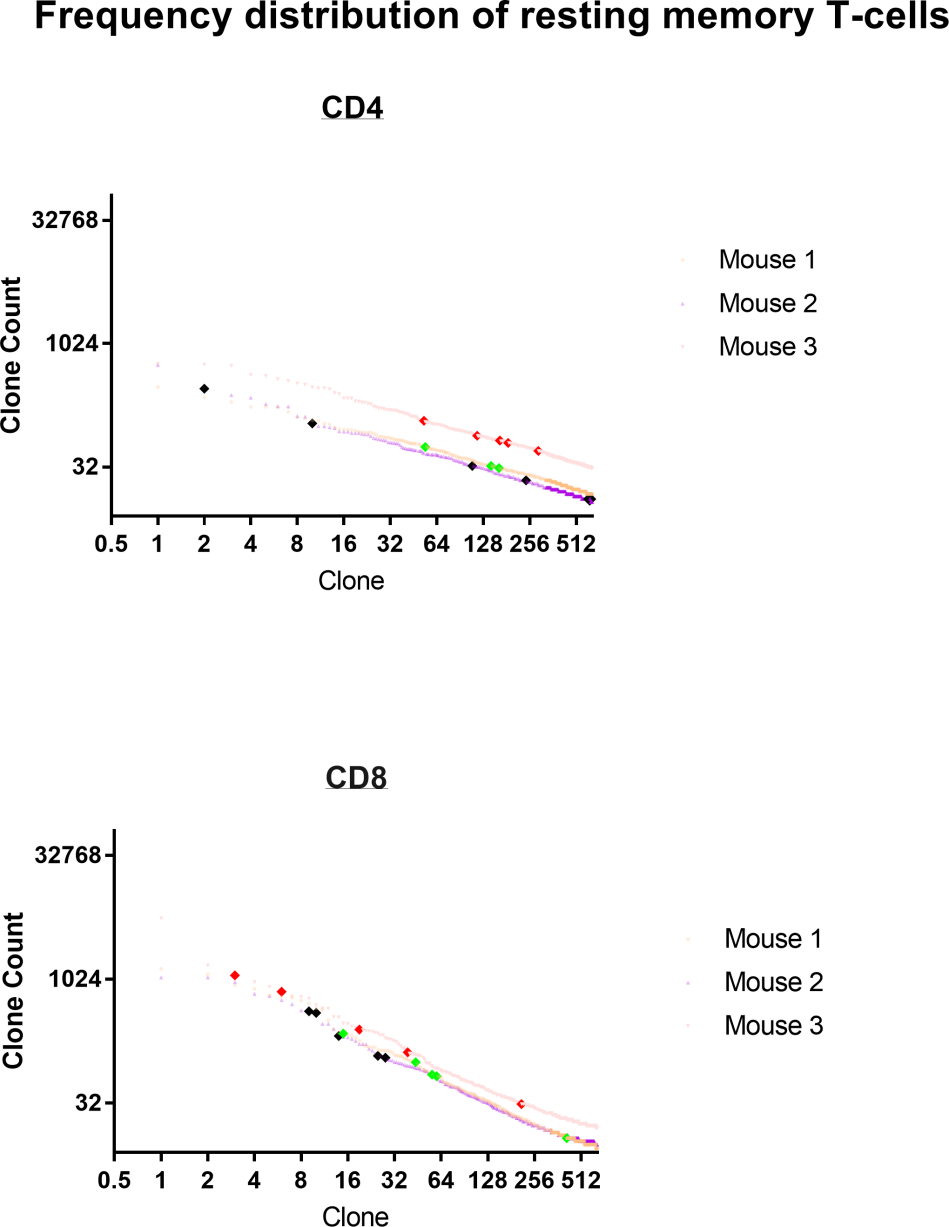
Frequency distribution of resting anti-tumor memory T-cells. The top 5 most frequent memory anti-tumor CD4 and CD8 TRB clonotypes from 3 animals are highlighted by large bold red, green or black diamonds within the frequency distribution of all resting spleen cells from the same animal.

Frequency analysis of resting T-cells does not distinguish anti-tumor memory T-cells from background (**Figure 14** for TRB and **Supplemental Figure S7** for TRA). The top 5 most frequent CD4 and CD8 TRA clonotypes from 3 animals are highlighted by large bold red, green or black diamonds within the frequency distribution of all resting spleen cells from the same animal. A stimulation is required to identify and quantify anti-tumor memory T-cells (**Figures 1**, **6**, **9**, **11**). In this sample size of 30, potential resting anti-tumor memory T-cell clones, 28 were found within the top 625 clones. As expected, following initial expansion, these resting memory cells did not diminish to rare frequency but neither did they stand out from the mixed background of resting frequencies.

## Discussion

This study found that curative viral onco-immunotherapy generated an oligoclonal anti-tumor memory response that could be quantified by bulk TCR clonal analysis following antigen stimulation. Frequency analysis of resting T-cells cannot distinguish these anti-tumor memory T-cells from background but antigen stimulation increases their frequency 10-fold, on average, in spleen and clearly identifies the most numerous of these cells. The high frequency T-cell clonotypes were higher than the highest control value for 100% of CD8 T-cells and 80% of CD4 T-cells showing that an effective anti-tumor response was identifiable in all animals. Multiple lines of evidence indicated that the high frequency clones were anti-tumor memory T-cells. First and most directly, one third of the high frequency CD8 T-cells were also identified by a known anti-tumor CD8 tetramer. Second, the high frequency spleen CD4 and CD8 T-cells were often identical to the peritoneal T-cells which have previously been shown to be anti-tumor memory T-cells. Third, transfer experiments showed that the same transferred clonotypes which cured tumors in host animals displayed high frequencies following stimulation in donor animals. Previous work with viruses and neoplasms, such as HIV and melanoma, has also used TCR clonotyping to track the anti-tumor and anti-virus T-cell response over time (26–31).

The total number of unique high-frequency anti-tumor memory clones can only be approximated from this data. Tetramer analysis, a direct measure of anti-tumor T-cells, found 2-4 (median = 3) peritoneal CD8 T-cell clones per animal. This is a minimum estimate because only a single antigen was interrogated. A second method, using a stringent standard that labeled as memory anti-tumor only clonotypes that reached a higher frequency following stimulation than any control values, found that unique clonotypes varied from 0-18 CD8 and 0-5 CD4 T-cell TRA or TRB anti-tumor clonotypes per animal (median CD8 = 2.5 and CD4 = 1.5). This estimate is also certainly low because some tetramer positive CD8 T-cells did not meet this stringent criteria. Effective anti-tumor clones that have low initial resting frequency or those that are less responsive to tumor antigen presented in the format used in this model system might not reach the stringent threshold values. A third method analyzing donor memory cells that cured tumor in host animals classified peritoneal cells as definite, active anti-tumor memory if they appeared in high frequency following stimulation in both donor and host. The number of unique anti-tumor memory clonotypes in the peritoneum varied from 1 to 8 in CD4 and 4-8 in CD8 T-cells with a median of 4 CD4 T-cells and 6 CD8 T-cells. Donor spleen cells were classified as anti-tumor memory if they appeared in high frequency following stimulation in both donor spleen and host peritoneum. The number of high-frequency unique anti-tumor memory TRB clonotypes in the donor spleen varied from 1 to 8 for CD4 and 5-7 for CD8 T-cells with a median of 3 CD4 T-cells and 6 CD8 T-cells. Overall, it is clear that stimulation elicits a high-frequency oligoclonal anti-tumor response of at least 1 CD4 and 3 CD8 memory T-cell clones. Memory CD4 T-cells are certainly present and necessary for an effective anti-tumor response, as shown in our previous work (4, 5, 10). The results are not comprehensive because sampling was obtained at only 2 tissue sites, at only one time point and with predominantly indirect identification of anti-tumor memory T-cells. In this study, anti-tumor memory CD8 T-cells were more frequently identified than CD4 T-cells but lymph node or blood may be a better tissue to sample for these cells than spleen or peritoneum. A more direct methodology to identify unique anti-tumor memory clones is required for a more precise count. Single cell RNA sequence analysis can add breadth by identifying unique TRA/TRB combinations as well as multiple receptor and transcription markers but is currently much more expensive than bulk TCR analysis (32, 33).

The resting frequency of memory CD4 T-cells (mean = 0.028%) and CD8 T-cells (mean = 0.11%) was low but still usually within the top 625 clones in each animal, as expected for memory T-cells (34). Stimulation produced a minimum of 6 doublings in 5 days with doublings in individual clones varying from 2 to 8. These doubling rates in memory T-cells closely match previous work assessing anti-virus memory T-cells in mice (22, 35–37). This response rate provides an effective immune response to tumor cells, which grow much more slowly than microorganisms, as shown by the consistent ability of cured animals to resist tumor rechallenge and of transferred T-cells to cure established implanted tumors (4, 5, 10, 16).

Tetramer analysis and stimulation assays showed that the oligoclonal high-frequency anti-tumor memory T-cells consisted predominantly of private clonotypes unique to each animal (7, 13, 38, 39). This result is not surprising because, as our tetramer data shows, many different clonotypes, even within a single animal, can create a set of CD8 T-cells with the same antigen specificity (13, 40). The number of unique clonotypes in the naïve T-cell population is extremely large and clonotypes expressed on just a small number of T-cells can expand to generate effective, permanent memory T-cells (18, 34). Previous studies have shown that the human CMV and Influenza virus T-cell responses also involve mainly private TCR clones but some public clones or clones biased to particular variable-gene usage are found and might be used to identify an anti-viral response in a population (41–45). Our data in a mouse tumor model of limited sample size hints that it will be difficult to find a set of high-frequency clonotypes that can be used to identify anti-tumor memory T-cells in a population. However, private clonotypes, once identified in an individual, can be tracked over time and location, in that individual. This study shows that these anti-tumor clonotypes can be identified initially by a very high-frequency response to tumor antigen stimulation.

Tetramer analysis confirmed that clonotype frequency determined by T-cell receptor (TCR)-β (TRB) analysis closely approximated cell clone frequency determined by flow cytometry. Clonotype frequency depends on the number of mRNA molecules per cells and efficiency of PCR amplification as well as the number of T-cells expressing the clonotype but these factors did not greatly alter the correspondence between cell and clonotype frequency in this experimental system (33). Clonotype analysis has the advantage that it can approximate the number of unique memory T-cell clones and their frequency without requiring knowledge of the specific tumor antigens. TRB analysis was superior to TRA analysis in estimating clone frequency perhaps because T-cells almost invariable express a single TRB mRNA but ~30% express 2 TRA mRNA (14, 15) and because the main contribution to TCR-peptide binding comes from the TRB CD3 sequence (25).

The methodology described in this report can be used immediately in pre-clinical work in mice to quantify the amplitude and diversity of the anti-tumor memory response in spleen to vaccination or treatment. Clinical application, however, will be confounded by a circulating repertoire in humans enriched in virus-reactive specificities (31, 42, 43) and requires further work to develop a practical method of stimulation in humans that produces a clear, acute response of anti-tumor T-cells in blood. Repeated blood testing is practical in humans and allows testing before and after stimulation, which will create a more sensitive test that can detect not only clonotypes with a large absolute oligoclonal response but also clonotypes that show a 10-fold increase following stimulation. Repeated blood testing will also allow following unique anti-tumor memory T-cell clonotypes in an individual over time.

## Data availability statement

The data presented in this paper are deposited with the NCBI BioProject with BioProject ID: PRJNA930664 and Submission ID: SUB12529129 and release date of 2023-03-17.

## Ethics statement

The animal study was reviewed and approved by Institutional Animal Research and Care Committee Protocol #: 21028761.

## Author contributions

YG is credited with contributing to the development of the idea for the article, as well as designing and performing experiments, preparing TCR data for analysis, analyzing all data and editing the manuscript. IB is credited with contributing to the development of the idea for the article, designing experiments, analyzing all data, and writing the paper. All authors contributed to the article and approved the submitted version.

## Supplementary material

Supplementary Figure 1 Frequency analysis of tumor-challenged peritoneal T-cells. Intraperitoneal tumor challenge in mice who had received rrVSV oncolytic immunotherapy to cure implanted tumor (tumor-cured mice) compared to mice with with full viral therapy but no tumor implant (virus control mice). Peritoneal CD4 and CD8 T-cells were harvested 5 days after challenge and TCR clones quantified. The top 100 clones are plotted and results shown separately for CD4 and CD8 T-cells and TCR-α (TRA) and TCR-β (TRB) receptors (Log2 scale for X and Y axes).

Supplementary Figure 2 Distinguishing true-positive tetramer+ clonotypes from contaminants in the sample mixture. High-frequency clonotypes (>1%) in individual animals were the index cases and the inverse ratio of the frequency of this clonotype in the individual mouse compared with the same clonotype in the tetramer+ mix was plotted.

Supplementary Figure 3 Comparison of the high frequency clonotypes following challenge in donor animals cured of implanted tumor by rrVSV oncolytic immunotherapy and host animals cured by T-cell transfer from the donor animals. Graphs show CD4 and CD8 T-cell TRA data from the same representative pair of animals of three total pairs shown in Fig. 9. The top 10 most frequent clones in the host peritoneal cells are the index clones and are compared with the donor peritoneal and spleen cells and the host spleen and blood cells. Identical numbers and colors within the CD4 and CD8 sets refer to identical clonotypes.

Supplementary Figure 4 Identifying and determining the frequency of resting and challenged anti-tumor memory T-cells. The top 5 most frequent clones in the host challenged peritoneal T-cells are the index clones and are compared with the frequency of the same clones in the spleens of challenged host and unchallenged donor mice. Three trios of animals are shown in separate rows with the CD4 T-cells in the top half and the CD8 T-cells in the bottom half of the figure (TRA only). Identical numbers and colors within the CD4 and CD8 pairs refer to identical clonotypes.

Supplementary Figure 5 Identifying and determining the frequency of resting and challenged anti-tumor memory T-cells. The frequency of the top 5 anti-tumor memory T-cells in the peritoneum of host challenged mice are compared with the frequency of the same clones in the spleen of host challenged mice and the spleen of resting donor mice. (n=3 in each group, CD4 and CD8, TRA only); mean values and SEM bars above the column for each group).

Supplementary Figure 6 Response to stimulation of individual anti-tumor memory T-cell clones. The total number of memory T-cells in transferred donor spleen cells were compared with total number of stimulated host memory T-cells harvested from spleen and peritoneum. The top 5 most frequent CD4 and CD8 memory T-cell clones for each of 3 animals are plotted separately (TRA clonotypes). The mean increase in clone count was 41-fold for CD4 and 67-fold for CD8 T-cells.

Supplementary Figure 7 Frequency distribution of resting anti-tumor memory T-cells. The top 5 most frequent CD4 and CD8 TRA clonotypes from 3 animals are highlighted by large bold red, green or black diamonds within the frequency distribution of all resting spleen cells from the same animal.
