# Supplementary figures and images for: Anti-tumor memory CD4 and CD8 T-cells quantified by bulk T-cell receptor (TCR) clonal analysis
Source: Front Immunol. 2023 Mar 23;14:1137054. doi: 10.3389/fimmu.2023.1137054 (PMC10076582; doi:10.3389/fimmu.2023.1137054)

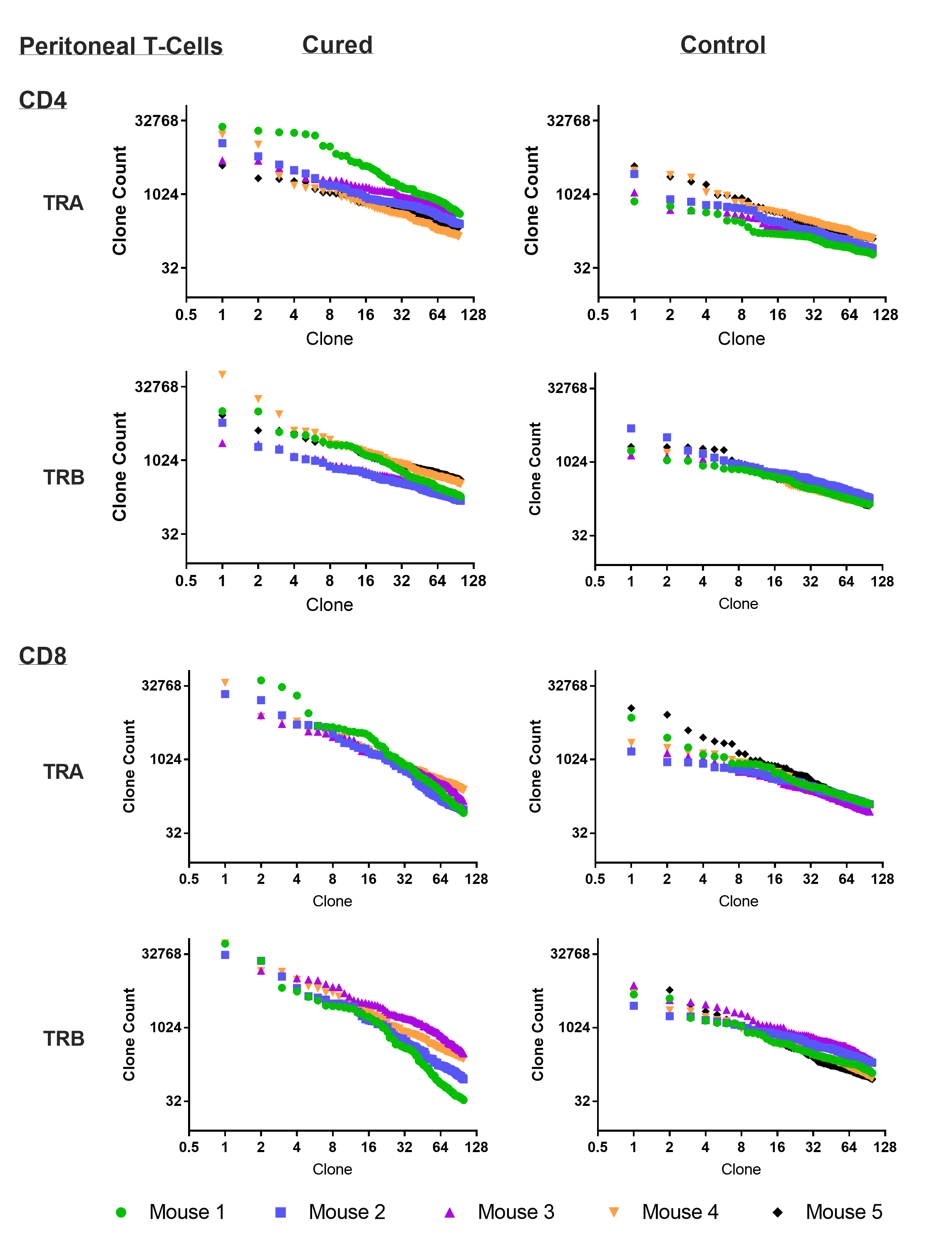

Supplement: Supplementary Figure 1 — Frequency analysis of tumor-challenged peritoneal T-cells. Intraperitoneal tumor challenge in mice who had received rrVSV oncolytic immunotherapy to cure implanted tumor (tumor-cured mice) compared to mice with with full viral therapy but no tumor implant (virus control mice). Peritoneal CD4 and CD8 T-cells were harvested 5 days after challenge and TCR clones quantified. The top 100 clones are plotted and results shown separately for CD4 and CD8 T-cells and TCR-α (TRA) and TCR-β (TRB) receptors (Log2 scale for X and Y axes). [file Image_1.tif]

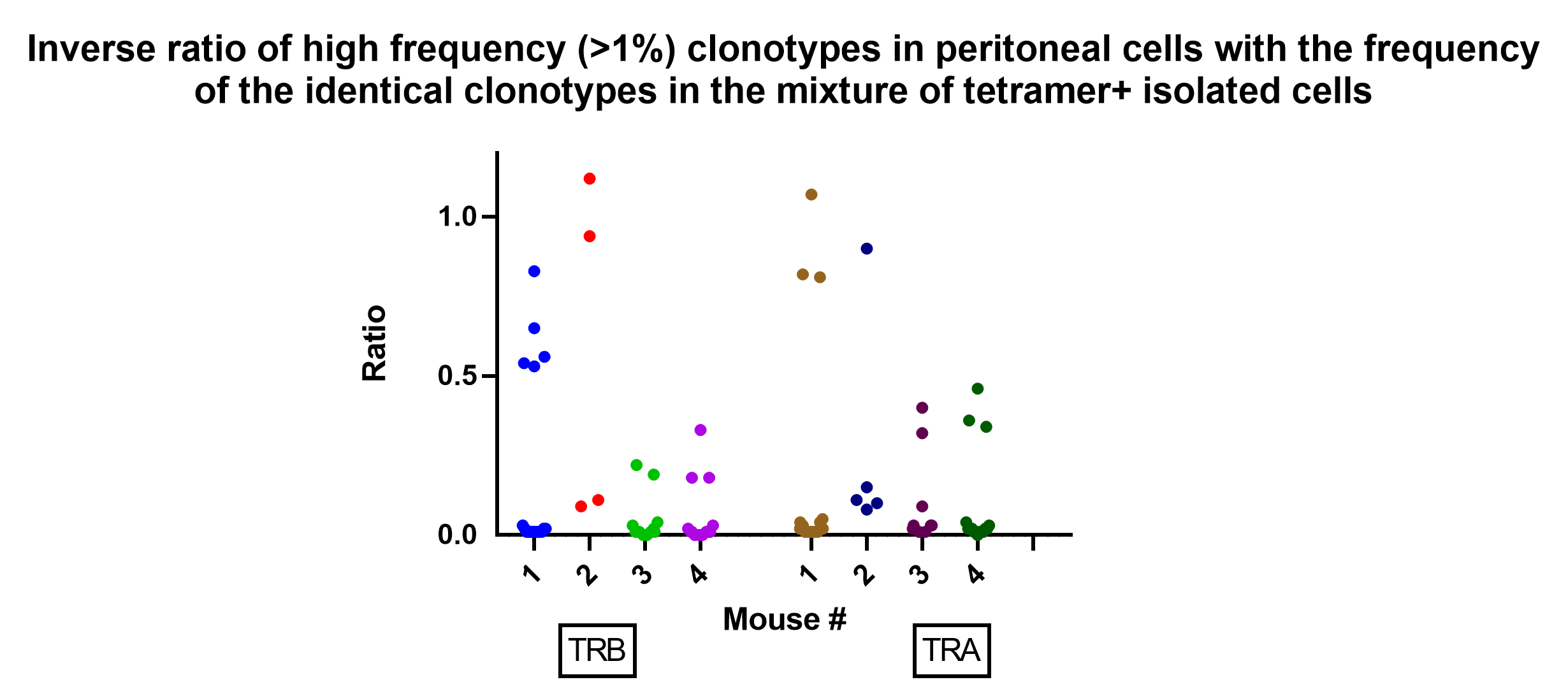

Supplement: Supplementary Figure 2 — Distinguishing true-positive tetramer+ clonotypes from contaminants in the sample mixture. High-frequency clonotypes (>1%) in individual animals were the index cases and the inverse ratio of the frequency of this clonotype in the individual mouse compared with the same clonotype in the tetramer+ mix was plotted. [file Image_2.tif]

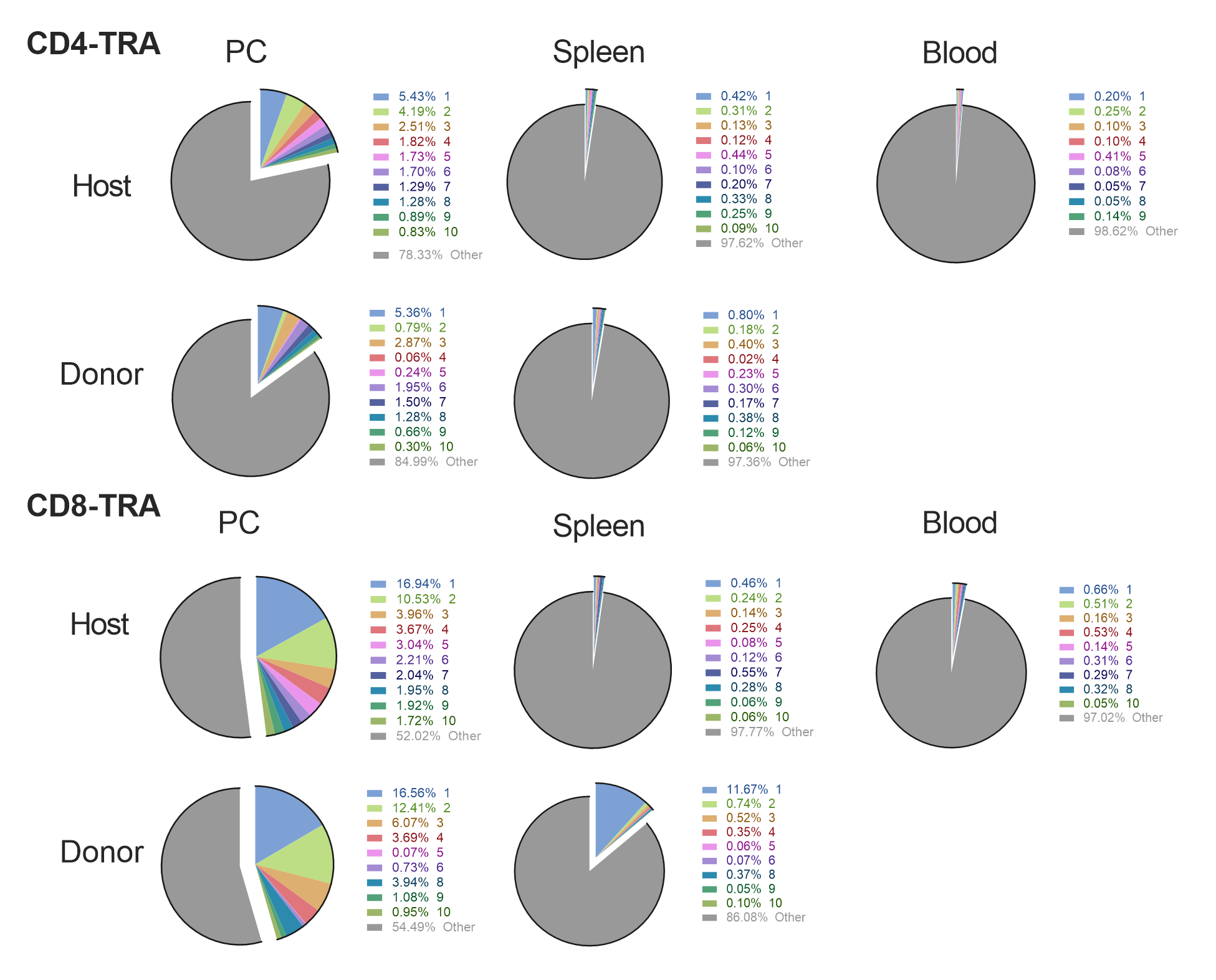

Supplement: Supplementary Figure 3 — Comparison of the high frequency clonotypes following challenge in donor animals cured of implanted tumor by rrVSV oncolytic immunotherapy and host animals cured by T-cell transfer from the donor animals. Graphs show CD4 and CD8 T-cell TRA data from the same representative pair of animals of three total pairs shown in Fig. 9. The top 10 most frequent clones in the host peritoneal cells are the index clones and are compared with the donor peritoneal and spleen cells and the host spleen and blood cells. Identical numbers and colors within the CD4 and CD8 sets refer to identical clonotypes. [file Image_3.tif]

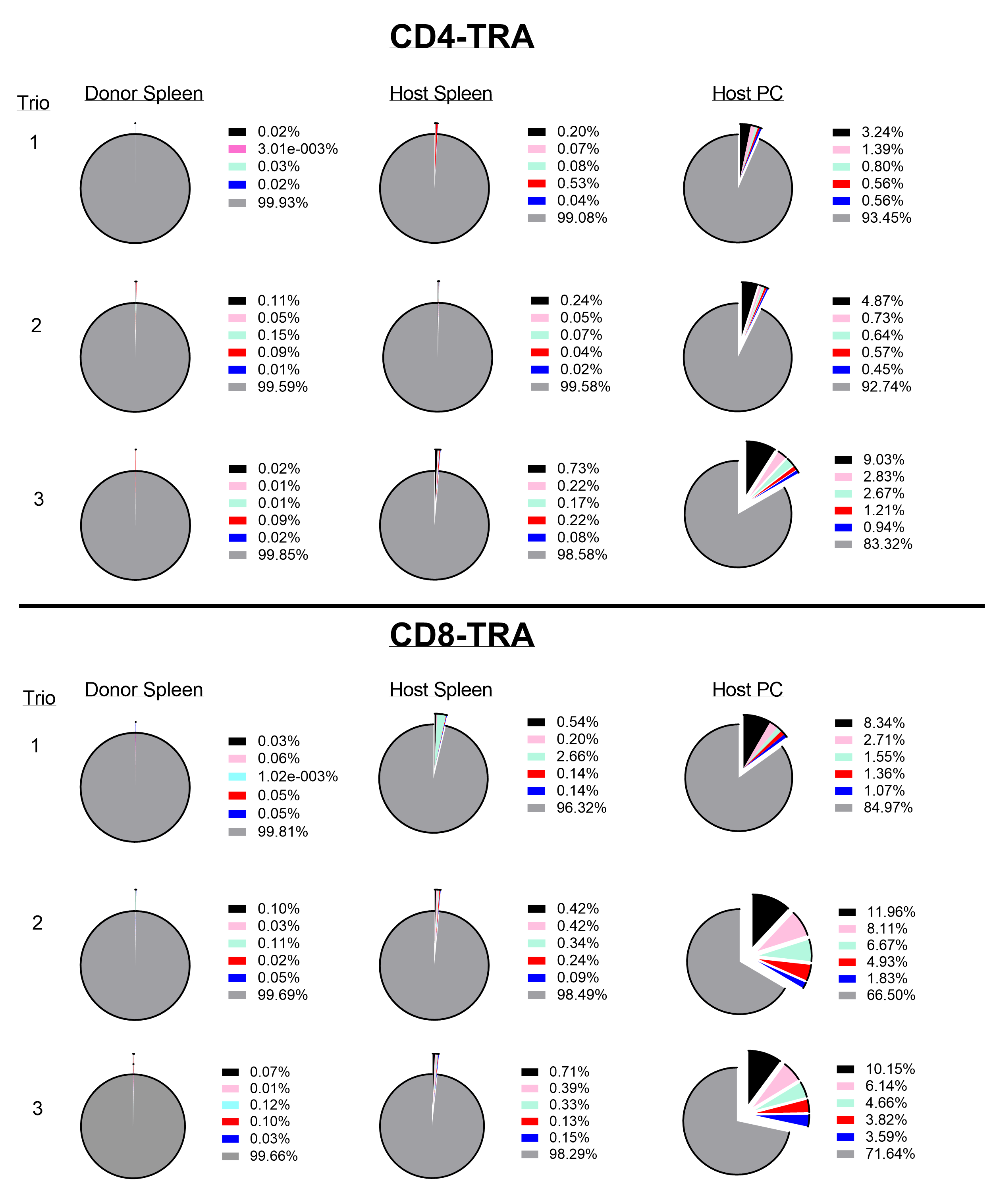

Supplement: Supplementary Figure 4 — Identifying and determining the frequency of resting and challenged anti-tumor memory T-cells. The top 5 most frequent clones in the host challenged peritoneal T-cells are the index clones and are compared with the frequency of the same clones in the spleens of challenged host and unchallenged donor mice. Three trios of animals are shown in separate rows with the CD4 T-cells in the top half and the CD8 T-cells in the bottom half of the figure (TRA only). Identical numbers and colors within the CD4 and CD8 pairs refer to identical clonotypes. [file Image_4.tif]

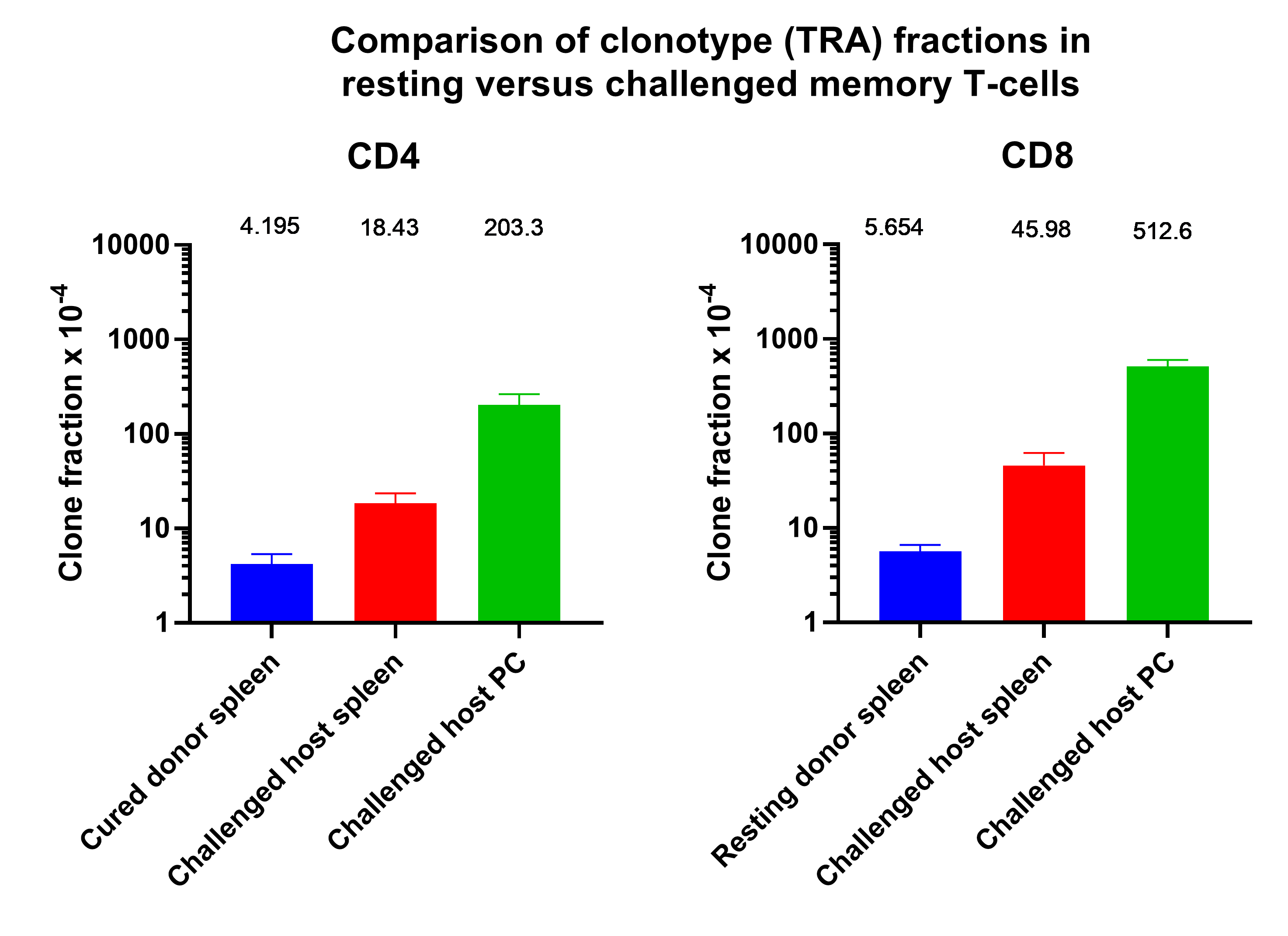

Supplement: Supplementary Figure 5 — Identifying and determining the frequency of resting and challenged anti-tumor memory T-cells. The frequency of the top 5 anti-tumor memory T-cells in the peritoneum of host challenged mice are compared with the frequency of the same clones in the spleen of host challenged mice and the spleen of resting donor mice. (n=3 in each group, CD4 and CD8, TRA only); mean values and SEM bars above the column for each group). [file Image_5.tif]

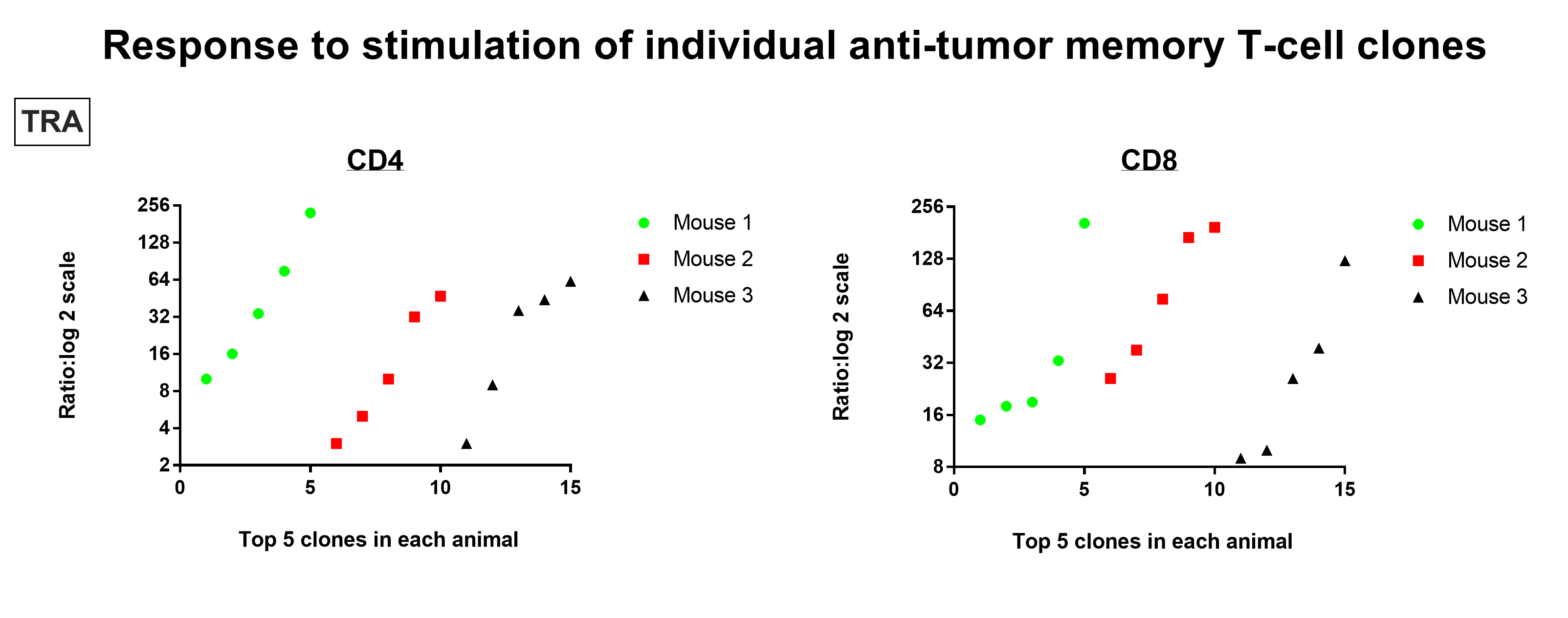

Supplement: Supplementary Figure 6 — Response to stimulation of individual anti-tumor memory T-cell clones. The total number of memory T-cells in transferred donor spleen cells were compared with total number of stimulated host memory T-cells harvested from spleen and peritoneum. The top 5 most frequent CD4 and CD8 memory T-cell clones for each of 3 animals are plotted separately (TRA clonotypes). The mean increase in clone count was 41-fold for CD4 and 67-fold for CD8 T-cells. [file Image_6.tif]

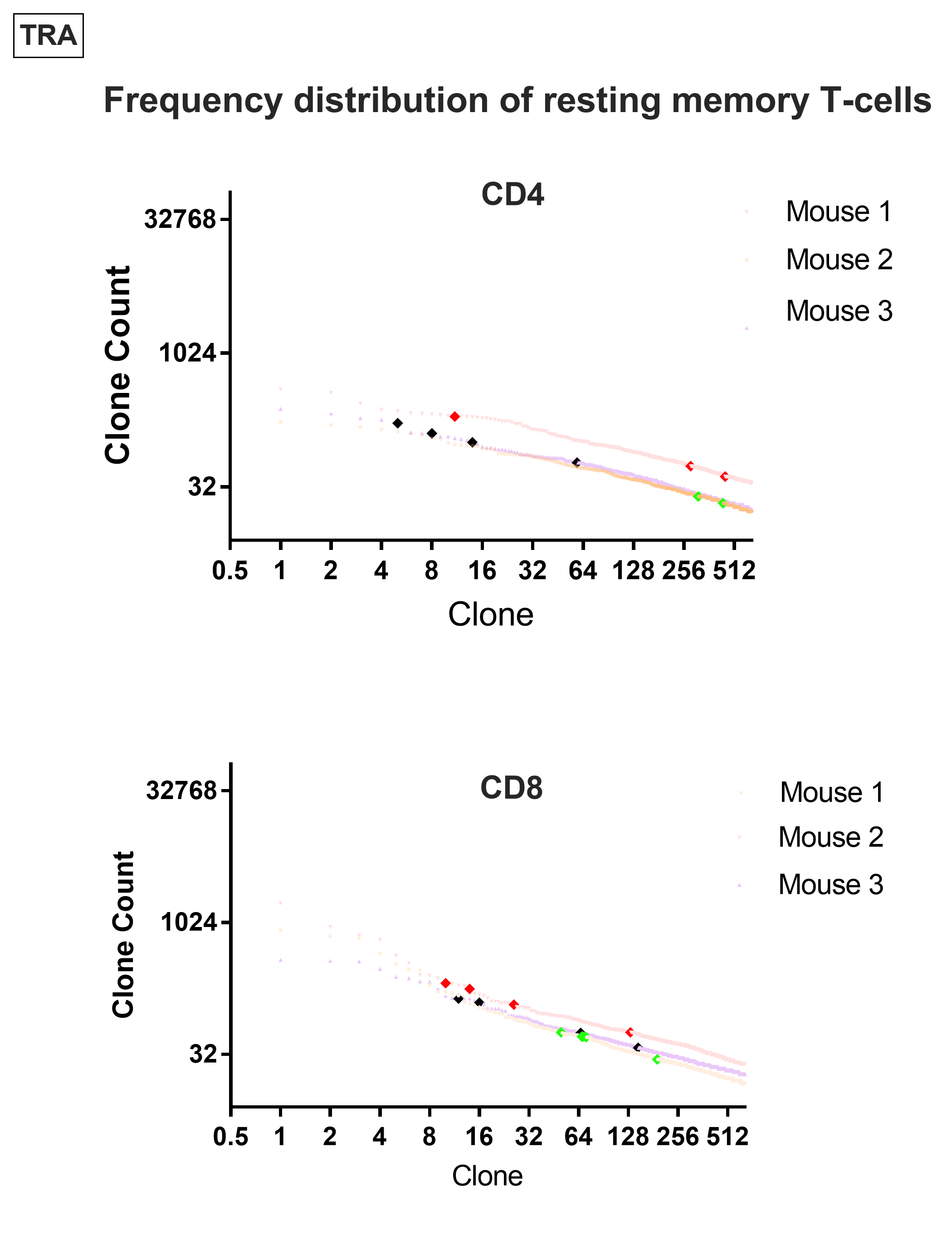

Supplement: Supplementary Figure 7 — Frequency distribution of resting anti-tumor memory T-cells. The top 5 most frequent CD4 and CD8 TRA clonotypes from 3 animals are highlighted by large bold red, green or black diamonds within the frequency distribution of all resting spleen cells from the same animal. [file Image_7.tif]
